# High-Throughput Sequencing-Based Investigation of Viruses in Human Cancers by Multienrichment Approach

**DOI:** 10.1093/infdis/jiz318

**Published:** 2019-06-28

**Authors:** Sarah Mollerup, Maria Asplund, Jens Friis-Nielsen, Kristín Rós Kjartansdóttir, Helena Fridholm, Thomas Arn Hansen, José Alejandro Romero Herrera, Christopher James Barnes, Randi Holm Jensen, Stine Raith Richter, Ida Broman Nielsen, Carlotta Pietroni, David E Alquezar-Planas, Alba Rey-Iglesia, Pernille V S Olsen, Ewa Rajpert-De Meyts, Line Groth-Pedersen, Christian von Buchwald, David H Jensen, Robert Gniadecki, Estrid Høgdall, Jill Levin Langhoff, Imre Pete, Ildikó Vereczkey, Zsolt Baranyai, Karen Dybkaer, Hans Erik Johnsen, Torben Steiniche, Peter Hokland, Jacob Rosenberg, Ulrik Baandrup, Thomas Sicheritz-Pontén, Eske Willerslev, Søren Brunak, Ole Lund, Tobias Mourier, Lasse Vinner, Jose M G Izarzugaza, Lars Peter Nielsen, Anders Johannes Hansen

**Affiliations:** 1 Centre for GeoGenetics, Natural History Museum of Denmark, University of Copenhagen, Denmark; 2 Department of Bio and Health Informatics, Technical University of Denmark, Lyngby, Denmark; 3 Novo Nordisk Foundation Center for Protein Research, Faculty of Health and Medical Sciences, University of Copenhagen, Denmark; 4 Department of Growth and Reproduction, Copenhagen University Hospital (Rigshospitalet), Denmark; 5 Department of Pediatrics and Adolescent Medicine, University Hospital Rigshospitalet, Denmark; 6 Department of Otorhinolaryngology, Head and Neck Surgery and Audiology, Rigshospitalet, Copenhagen University Hospital; 7 Department of Dermato-Venerology, Faculty of Health Sciences, Copenhagen University Hospital, Bispebjerg Hospital, Denmark; 8 Department of Pathology, Herlev and Gentofte Hospital, University of Copenhagen, Denmark; 9 National Institute of Oncology, Department of Gynecology, Budapest, Hungary; 10 1st Department of Surgery, Semmelweis University, Budapest, Hungary; 11 Department of Clinical Medicine, Aalborg University, Denmark; 12 Department of Haematology, Aalborg University Hospital, Denmark; 13 Department of Pathology, Aarhus University Hospital, Denmark; 14 Department of Clinical Medicine, Department of Haematology, Aarhus University Hospital, Denmark; 15 Department of Surgery, Herlev and Gentofte Hospital, University of Copenhagen, Denmark; 16 Center for Clinical Research, North Denmark Regional Hospital and Department of Clinical Medicine, Aalborg University, Hjørring, Denmark; 17 Centre of Excellence for Omics-Driven Computational Biodiscovery, AIMST University, Kedah, Malaysia; 18 Department of Autoimmunology and Biomarkers, Statens Serum Institut, Copenhagen S, Denmark

**Keywords:** cancer, enrichment, human, virome

## Abstract

**Background:**

Viruses and other infectious agents cause more than 15% of human cancer cases. High-throughput sequencing-based studies of virus-cancer associations have mainly focused on cancer transcriptome data.

**Methods:**

In this study, we applied a diverse selection of presequencing enrichment methods targeting all major viral groups, to characterize the viruses present in 197 samples from 18 sample types of cancerous origin. Using high-throughput sequencing, we generated 710 datasets constituting 57 billion sequencing reads.

**Results:**

Detailed in silico investigation of the viral content, including exclusion of viral artefacts, from de novo assembled contigs and individual sequencing reads yielded a map of the viruses detected. Our data reveal a virome dominated by papillomaviruses, anelloviruses, herpesviruses, and parvoviruses. More than half of the included samples contained 1 or more viruses; however, no link between specific viruses and cancer types were found.

**Conclusions:**

Our study sheds light on viral presence in cancers and provides highly relevant virome data for future reference.

## Teaser

High-throughput sequencing of approximately 200 cancer samples detected viruses from 7 viral families. More than half of the investigated samples contained 1 or more viruses; however, no associations linking specific viruses with specific cancer types were found.

Globally, more than 15% of human cancer cases occurring in 2008 could be ascribed to infectious agents classified as carcinogenic according to the International Agency for Research on Cancer (IARC) [1]. This excludes viruses and cancer sites for which evidence of carcinogenicity is weaker. The IARC-classified carcinogenic agents include 6 types of viruses: hepatitis B and C virus, high-risk human papillomaviruses (HPVs), human herpesvirus (HHV) 4 (Epstein-Barr virus), human T-cell lymphotropic virus type, and HHV 8 (Kaposi’s sarcoma-associated herpesvirus). Hepatitis B virus-associated hepatocellular carcinoma and HPV-associated cervical and anal cancer can be prevented through vaccination [2, 3]. Apart from both firmly and less firmly established associations, additional cancers might be caused by either known or unknown viruses and could therefore be preventable.

With the introduction of high-throughput sequencing, description of the virome of various tissues of both healthy and diseased individuals has accelerated [4–13], generating important knowledge about the viral species hosted by humans. Application of high-throughput sequencing led to the discovery of Merkel cell polyomavirus (MCPyV) suspected of causing Merkel cell carcinomas [14], and, in later years, large-scale investigations of viral expression in high-throughput ribonucleic acid (RNA)-sequencing data and of viral sequences in whole genomes or exomes based on data from The Cancer Genome Atlas have been conducted [15–17]. These studies have confirmed established virus-cancer associations and raised questions about hypothesised associations, but thus far they have not revealed novel associations.

Infection with carcinogenic viruses is common but only rarely leads to cancer. Upon transformation, the virus persists intracellularly as an episome or is integrated in the host cell genome [18]. To target the multiple possible types and stages of viral genomes, we applied sensitive presequencing methods for enrichment of virions [19], enrichment of circular deoxyribonucleic acid (DNA) genomes [20], and for capturing retroviral [21] or vertebrate viral sequences [22]. The methods were applied, along with high-throughput sequencing of total DNA and RNA, to 197 samples from 18 cancer types (including biopsies, bone marrow, and urine samples) as well as samples of ascites, blood from colon cancer patients, and a few healthy control samples. Targeting a breadth of viruses, we present a comprehensive characterization of the virome of the included cancer samples, thus expanding the reference catalog of the viruses found in these cancers.

## METHODS

### Samples and Datasets

The present study includes 760 datasets generated from 197 patient samples and 50 nontemplate controls. Some of the datasets were included in previous studies (see Supplementary Methods). Viral sequence contamination in the included samples is explored in detail in a separate study [23]. The description of all samples and laboratory and bioinformatic methods applied are provided here for the sake of completeness.

### Ethics Statement

Human sample collection, handling, and analysis were performed under ethical protocol H-2-2012-FSP2 (Regional Committee on Health Research Ethics) and case no. 1304226 (National Committee on Health Research Ethics). In accordance with National legislation (Sundhedsloven), all human samples were processed anonymously.

### Patient Samples

All samples are listed in Table 1. Detailed information regarding samples and datasets can be found in Supplementary Methods and Supplementary Table S1.

**Table 1.** Samples and Datasets Included in the Study

					Virion Enrichment			Capture				
Sample Type	Sample Material	Samples (n)	Total DNA	Total RNA	DNA	RNA	Circular DNA Enrichment	Retrovirus DNA	Retrovirus mRNA	Vert. Virus DNA	mRNA	Datasets (n)
Basal cell carcinoma (cutaneous)	Tumor biopsies	11	11		11	11	4	6		11		54
Mycosis fungoides (cutaneous)	Tumor biopsies	11	11		11	11	10	10		11		64
Melanoma (cutaneous)	Tumor biopsies	10	10		10	10	8			10		48
Oral cancer	Tumor biopsies	10	9		10	10	10			10		49
Oral healthy	Healthy tissue	1					1			1		2
Vulvar cancer	Tumor biopsies	3			3	3	3			3		12
Bladder cancer	Tumor biopsies	7			7	7	5			7		26
Bladder cancer urine	Urine	10		2			10			4		16
Colon cancer	Tumor biopsies	16	12	11	3	3		6			6	41
Colon healthy	Healthy tissue	2									2	2
Breast cancer	Tumor biopsies	20	20	19	17	20	15					91
Testicular cancer	Tumor biopsies	20	5		20	20						45
AML	Bone marrow (sorted cells)	9		6	9	9	7					31
B-CLL	Blood/bone marrow (sorted cells)	9		8	9	9	8	9		8		51
BCP-ALL	Bone marrow	8			8	8	8					24
CML	Bone marrow (sorted cells)	10		10	10	10	10			10		50
T-ALL	Bone marrow (nonsorted/sorted cells)	11		9	11	11	9					40
DLBCL	Cell lines	5	5						3		3	11
Lymphoblastic lymphoma	Cell lines	1	1						1		1	3
Multiple myeloma	Cell lines	6	6						2		2	10
Colon cancer blood	Blood	8	8									8
Colon cancer ascites	Ascites	1	1					1				2
Breast cancer ascites	Ascites	1	1	1	1	1	1					5
Ovarian cancer ascites	Ascites	5	5	4	3	3	5					20
Pancreatic cancer ascites	Ascites	2	2	2				1				5
NTC					19	18	5	1		7		50
Total (without NTC)		197	107	72	143	146	114	33	6	75	14	710

Abbreviations: AML, acute myeloid leukaemia; B-CLL, B-cell chronic lymphocytic leukaemia; BCP-ALL, B-cell precursor acute lymphoblastic leukaemia; CML, chronic myelogenous leukaemia; DLBCL, diffuse large B-cell lymphoma; DNA, deoxyribonucleic acid; mRNA, messenger ribonucleic acid; NTC, nontemplate control; RNA, ribonucleic acid; T-ALL, T-lineage acute lymphoblastic leukaemia; Vert., vertebrate.

### Total Deoxyribonucleic Acid Analysis

Total DNA was extracted using the QIAamp DNA Mini kit (QIAGEN). The DNA libraries were prepared from 1 μg of DNA using either the TruSeq DNA protocol (PE-940-2001) (Illumina) or an in-house protocol [24] using NEBNext reagents (E6070) (New England BioLabs).

### Total Ribonucleic Acid (RNA) and Messenger RNA Analysis

Total RNA was extracted using the High Pure Viral RNA kit (Roche), RNeasy Mini Kit (QIAGEN), or QIAamp DNA Mini Kit. Messenger RNA (mRNA) was extracted using Dynabeads mRNA Direct Purification Kit (Invitrogen). The RNA libraries were prepared using ScriptSeq v2 RNA-Seq or ScriptSeq Complete Gold Library Preparation Kit (Epicentre). See Supplementary Methods for details regarding extraction kits, ribosomal RNA depletion, and library preparation kits used.

### Circular Deoxyribonucleic Acid Enrichment

Enrichment of small circular DNA molecules was performed on total DNA extracts based on phi29 DNA polymerase-mediated amplification of exonuclease-treated extracts as previously described with minor modifications [20]. Two micrograms of DNA was fragmented using the Bioruptor NGS (Diagenode) to an average length of 300 base pairs (bp). Libraries were prepared as described for total DNA analysis.

### Retrovirus Capture

Two versions of retrovirus capture were applied. Retrovirus capture v1 includes 118 retroviral reference sequences [21] (Supplementary Table S2). Capture was performed with 1 μg of single indexed libraries prepared from total DNA or mRNA (see above) according to the SeqCap EZ library SR protocol (Roche NimbleGen) (capture dataset numbers between s0001 and s1112 [Supplementary Table S1]). Retrovirus capture v2 includes 98 retroviral reference sequences (Supplementary Table S2). Capture was performed with 500 μg of double-indexed libraries prepared from total DNA according to the MYcroarray MYbaits protocol version 2.3.1 with some modifications according to protocol version 1.3.8 (separating the beads from the eluted captured library and addition of neutralization buffer to the supernatant) (capture dataset numbers s1431–s1440 [Supplementary Table S1]).

### Vertebrate Virus Capture Deoxyribonucleic Acid

The vertebrate virus capture probe design includes 2339 sequences representing viral species found in vertebrates, excluding fish [22] (Supplementary Table S2). Sequences representing (MCPyV), KI polyomavirus, and HHV5 were not included in genomes used for probe design. SeqCap EZ hybridization probes were designed and synthesized by Roche NimbleGen. Capture was performed on double-indexed libraries prepared from total DNA extracted using DNeasy Blood and Tissue (QIAGEN) or QIAamp DNA Mini kit. Libraries were prepared as described for total DNA analysis. Viral sequences were captured from 1 μg of pooled libraries as described in [22] with the following modifications: hybridization buffer without 10% formamide was used, and the amplified captured libraries were purified using QIAquick PCR Purification Kit (QIAGEN).

### Enrichment of Virion-Associated Deoxyribonucleic Acid and Ribonucleic Acid 

Samples used for enrichment were fresh frozen after collection with no addition of nucleic acid preservers. Enrichment was performed as previously described [25]. The DNA libraries were prepared using the Nextera or Nextera XT DNA Sample Preparation Kit (Illumina) and RNA libraries were prepared using ScriptSeq v2 RNA-Seq Library Preparation Kit (Epicentre), and both subsequently purified using the Agencourt AMPure XP PCR purification system (Beckman Coulter). In cases of insufficient amplification, libraries were reamplified using AccuPrime *Pfx* DNA polymerase (Life Technologies) and P5 and P7 primers.

### Sequencing and Data Analysis

Paired-end sequencing (2 × 100 bp) was performed on the Illumina HiSeq 2000 platform. The sequence analysis is detailed in the Supplementary Methods. In brief, reads were trimmed of adapter sequences and overlapping read pairs were merged. Human sequences were depleted by mapping to the human genome, and low-complexity regions were filtered out. De novo assembly was achieved using IDBA [26]. The reads and contigs were aligned to the NCBI nucleotide database (*nt*) using BLASTn (megablast) [27] with a cutoff e-value of 10^–3^. The best hit was defined based on highest bit-score. Regions in the contigs having no BLASTn hits were aligned against the NCBI nonredundant protein database (*nr*) using BLASTx or DIAMOND [28] with a cutoff e-value of 10^–3^. Individual reads for s1431–s1523 were not blasted.

### Investigation of Human Viral Hits

To exclude false positives, all BLAST/DIAMOND hits to human viruses were evaluated in silico and categorized as confirmed viral hits or artefacts (see Supplementary Methods). For the contigs, hits were evaluated manually by alignment using Geneious software or web-based reblast. For the reads, hits were evaluated by mapping to a database of 343 selected viral reference genomes. The alignments were visualized using Circos [29]. All plots were visually inspected. Hits arising from bleedover [30, 31] were removed from both mapping results and contigs. For the read mapping, a lower cutoff of 180 (205 for human immunodeficiency virus [HIV]) bases covered was applied.

### Co-occurrence Network

Co-occurrence patterns among species occurring in 4 or more samples were investigated by performing Spearman’s rank correlations and network inference on the read mapping data. Human papillomaviruses and anelloviruses unclassified at species level were evaluated at strain level. Such strains, occurring in fewer than 4 samples, were disregarded as well, leaving only 2 anellovirus strains unclassified at species level (here termed Unclassified Anellovirus 1 and 2). Nontemplate controls were also excluded. Correlations were performed in vegan [32], and the network was constructed using igraph [33]. Networks were visualized using Cytoscape (v.3.6.0) [34].

### Statistics

Comparison of the proportions of virus-positive samples was performed using Fisher’s exact test, with a significance level of 0.05. For the co-occurrence network, co-occurrences were considered significant when Spearman’s correlation coefficient was >0.20 (*P* < .05) [35].

### Data Availability

Sequencing data depleted of human sequences is deposited at the NCBI sequence read archive (BioProject accession no. PRJNA416252). According to Danish law, publication of human sequences is not permitted without consent, which cannot be obtained, because all samples were anonymized. The complete coding sequences of HPV strains CGG5-287s1382c000001 and CGG5-301s0532c000007 and 6 contigs representing shorter genome fragments of novel HPV types are uploaded to GenBank (accession numbers MG869604–MG869611).

## RESULTS

### Investigation of Human Viral Hits

**Figure 1.**
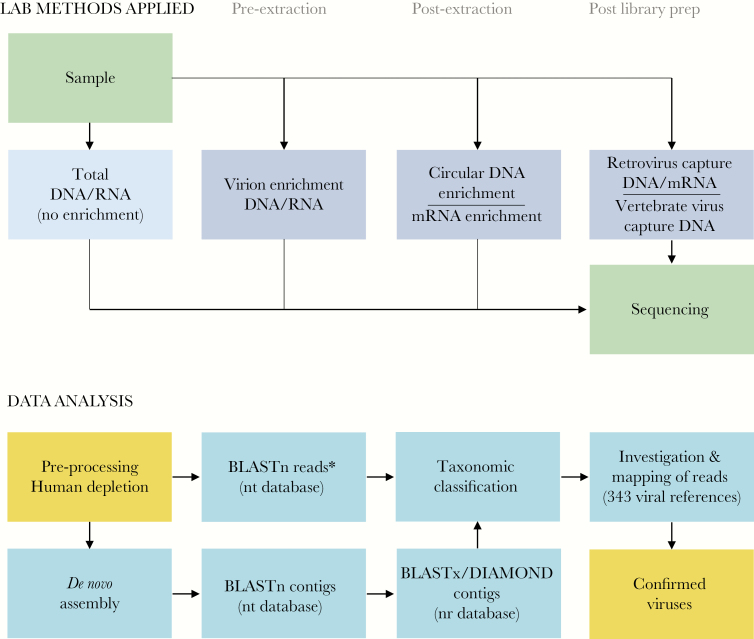
Laboratory methods and analysis pipeline. (Top) Schematic illustration of the laboratory methods used. Total deoxyribonucleic acid (DNA) or ribonucleic acid (RNA) was sequenced, or samples were exposed to one of the indicated enrichment methods before sequencing. (Bottom) Schematic illustration of the data analysis pipeline; de novo assembled contigs and human-depleted reads were analyzed with BLASTn and/or BLASTx/DIAMOND. Human viral hits were investigated in silico, and the reads were mapped to a database of selected viral reference genomes. *Applies to the majority of the datasets (see Methods).

We applied multiple viral enrichment methods (Figure 1) to 197 samples of diverse cancer types (Supplementary Table S1), resulting in 710 datasets (Table 1) and 50 nontemplate (negative) controls constituting >57 billion Illumina HiSeq read pairs, with the median number of reads per dataset ranging from 30.5 to 169 million, depending on the method applied (Supplementary Table S3). De novo assembly of the nonhuman fraction of the reads yielded ~1.5 million contigs. The taxonomy of contigs and reads was assigned using a BLAST-based pipeline (Figure 1 and Supplementary Material). These analyses are hereafter referred to as BLASTnx (for contigs) or BLASTn (for reads).

Investigation of the viral BLAST hits (Supplementary Table S4) revealed artefacts arising mainly due to short, local-only sequence similarity to viral genomes. Therefore, all hits to human viruses were evaluated in silico (see Supplementary Methods and Results). For the contigs, confirmed hits to 61 viruses from 6 viral families were found, whereas 14 human viruses were disregarded as false positives (Supplementary Table S4). For the reads, mapping to 343 manually selected viral genomes, hereafter referred to as read mapping, confirmed viral hits to 146 reference genomes (Supplementary Table S5; for mapping results, see Supplementary Data 1 and coverage plots in Supplementary Figure S1). The artefactual viral sequences identified in our data are explored further in a separate study [23]. Confirmed viral hits (Supplementary Table S6 and Supplementary Data 2) were further depleted of bleedover of viral reads occurring during sequencing.

### The Virome of the Cancerous Samples

**Figure 2.**
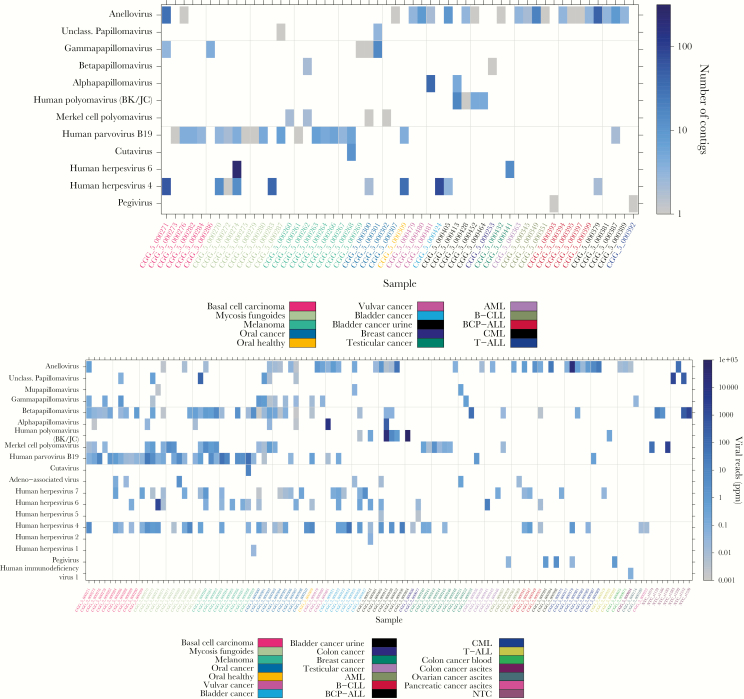
Viruses detected from BLASTnx of contigs and read mapping. (Top) The number of contigs detected across cancer types (horizontal axis), indicated by color (right legend). Only confirmed viral hits are included. (Bottom) The fraction of viral reads in parts per million (ppm) detected across cancer types (horizontal axis), indicated by color (right legend). Only confirmed viral hits are included. AML, acute myeloid leukemia; B-CLL, B-cell chronic lymphocytic leukaemia; BCP-ALL, B-cell precursor acute lymphoblastic leukaemia; CML, chronic myeloid leukemia; T-ALL, T-lineage acute lymphoblastic leukaemia. NTC, nontemplate control.

Of the 197 samples included, 54 (27%) were virus-positive at contig level, whereas 106 (54%) were virus-positive from read mapping. For several skin-associated and mucosal cancer types, all samples were found virus-positive (Supplementary Table S7), whereas certain sample types revealed no confirmed viral sequences. The detected viruses mainly belong to the families *Papillomaviridae*, *Polyomaviridae*, *Herpesviridae*, *Parvoviridae*, and *Anelloviridae*. Throughout the results, the identified viruses are grouped at species or genus level for both contig BLASTnx and read mapping (Figure 2), and the individual viral strains identified are presented fully in the Supplementary Material (Supplementary Figures S2 and S3). Between 2 and 7 different viral genera were represented in the virus-positive samples (median of 2) (Supplementary Figure S4), with the highest diversity of viral genera generally occurring in skin-associated and mucosal cancers (Supplementary Table S9).

#### Papillomaviruses

**Figure 3.**
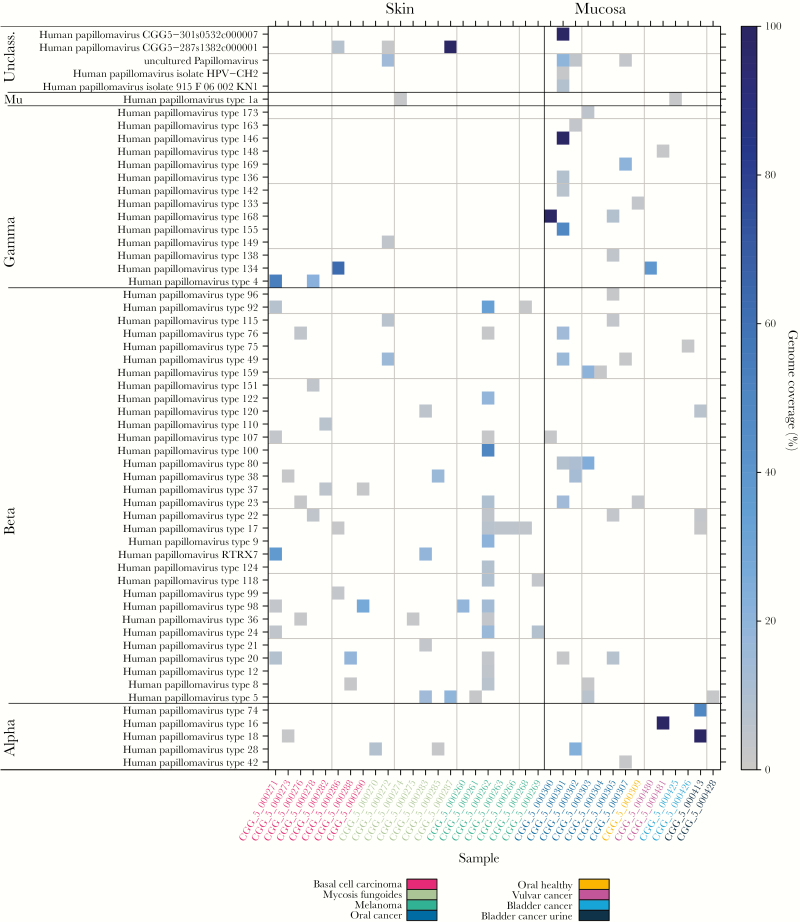
Human papillomaviruses (HPVs) identified in skin and mucosal cancers. Genome coverage (%) for the different HPV types found in samples of skin and mucosal cancers, indicated by color (right legend) (the full dataset is shown in Supplementary Figure S3). Only confirmed viral hits are included.

Human papillomaviruses were detected mainly in skin and mucosa-associated cancers (64%–73% of samples) (Table 2, Figures 2 and 3). De novo assembly recovered the full genome of a novel type of *Gammapapillomavirus* in a single contig in an oral cavity cancer sample (HPV strain CGG5-301s0532c000007 [Supplementary Table S10]), being most similar to HPV146 (Supplementary Figure S5 and Supplementary Methods). Contigs representing shorter genome fragments of novel HPV types and full genomes of known types were also detected (Supplementary Table S10). High-risk alphapapillomaviruses were found in a few samples; HPV16 and HPV18 in contigs (full genomes) and HPV18 and HPV42 from read mapping (at low coverage). The HPV-positive skin-associated and mucosal samples contained sequences mapping to up to 17 different HPV types (median, 2 types), with oral cavity cancers showing the highest numbers (median, 5 types) (see Discussion). In skin-associated cancers, *Betapapillomavirus* was the most represented genus (Figures 2 and 3, Supplementary Table S8), differing from previous studies of healthy skin [5, 9], whereas oral cavity cancers showed high *Betapapillomavirus* and *Gammapapillomavirus* positivity, also contrasting previous findings [9, 36].

**Table 2.** Virus-Positive Samples From the Read Mapping Analysis

Sample Type	Samples (n)	*Papillomaviridae*	*Polyomaviridae*	*Herpesviridae*	*Parvoviridae*	*Anelloviridae*	*Flaviviridae*	*Retroviridae*
Basal cell carcinoma	11	8	3	5	10	1		
Mycosis fungoides	11	7	6	8	9	2		
Melanoma	10	7	3	6	8	1		
Oral cancer	10	7	4	9	2	5		
Oral healthy	1	1		1	1			
Vulvar cancer	3	2			1	3		
Bladder cancer	7	2	1	6	2	3		
Bladder cancer urine	10	2	5	4	1	5		
Colon cancer	16			2				
Breast cancer	20	3	6	3	1	1		
Testicular cancer	20			2	1	2		
AML	9	1	1		1	1	1	
B-CLL	9	1		3		3		
BCP-ALL	8					1	2	
CML	10	1		3	1	7	1	
T-ALL	11		1	1			1	
Colon cancer blood	8					2		
Colon cancer ascites	1					1		1
Ovarian cancer ascites	5	1		1				
Pancreatic cancer ascites	2			1				
Total no. of samples		43	30	55	38	38	5	1
Total no. of sample types		13	9	15	12	15	4	1

Abbreviations: AML, acute myeloid leukemia; B-CLL, B-cell chronic lymphocytic leukaemia; BCP-ALL, B-cell precursor acute lymphoblastic leukaemia; CML, chronic myeloid leukemia; T-ALL, T-lineage acute lymphoblastic leukaemia.

Notes: The number of samples positive for a given viral family is shown for each sample type. Extended counts are shown in Supplementary Table S8. Only confirmed viral hits are included.

#### Polyomaviruses

Polyomaviruses were detected mainly in skin-associated and certain mucosal cancers (Table 2, Supplementary Table S8, Figures 2, Supplementary Figure S2 and S3). In bladder cancer urine, BK polyomavirus (BKV) (33%–98% coverage [Supplementary Table S6]) and JC polyomavirus (JCV) (99% coverage) were detected, whereas most of the remaining polyomavirus-positive samples contained MCPyV. One bladder cancer urine sample was found positive for both JCV (>10 million reads, 99% coverage) and BKV (59 reads, 8.4% coverage), the latter finding possibly arising due to sequence homology between these 2 viruses (see Supplementary Discussion). Merkel cell polyomavirus was only detected when applying virion enrichment DNA, and single-nucleotide polymorphisms were found to recur between positive datasets, suggesting a possible contamination (see Supplementary Discussion).

#### Herpesviruses

Human herpesvirus 1, HHV2, and HHV5 were detected in a few samples each, all at low coverage (up to 0.34%), whereas HHV4, HHV6, and HHV7 were more widespread (Figure 2, Supplementary Table S8). Human herpesvirus 4 was found mainly in certain skin and mucosa-associated cancers, whereas HHV6 was found mainly in malignant melanoma, and HHV7 was found mainly in bladder cancer, oral cavity cancer, and mycosis fungoides. Human herpesvirus 6B and HHV7 were of low coverage, except a sample of mycosis fungoides and testicular cancer showing higher HHV6A coverage (99% and 53%). Human herpesvirus 4 also showed higher genome coverage in certain samples (up to 69%). In all samples showing presence of HHV6A (Supplementary Figure S3), reads mapping to both HHV6A and HHV6B were detected, likely arising due to sequence homology between these 2 species (see Supplementary Discussion).

#### Parvoviruses

Human parvovirus B19 was mainly detected in skin-associated cancers (80%–91% of samples by read mapping, 32%–100% coverage [Figure 2, Supplementary Tables S6 and S8]). The recently described cutavirus of the genus *Protoparvovirus* [37] was detected from contigs and read mapping in one sample of malignant melanoma as presented earlier [38]. In addition, adeno-associated virus-2 was detected in a few samples.

#### Anelloviruses

Anelloviruses were detected in the contigs at highest prevalence in certain mucosal cancers and leukemias (Table 2, Figure 2). Full or near full genomes were detected among the contigs (Supplementary Table S11), some of these possibly representing novel anellovirus species. Contigs and reads mapping to different anelloviruses were often seen (Supplementary Figures S2 and S3); however, species- and/or strain-level identification of these might be less certain (see Discussion and Supplementary Material).

#### Rare Occurrences

A few viruses occurred only sporadically. The flavivirus human pegivirus (formerly GB virus C) was detected in 2 samples of B-cell precursor acute lymphoblastic leukaemia (BCP-ALL) and 1 sample each of T-lineage acute lymphoblastic leukaemia (T-ALL), acute myeloid leukemia (AML), and chronic myelogenous leukemia (CML) (2.1%–17% coverage [Supplementary Table S6]), whereas HIV-1 was detected in ascites from a colon cancer patient (11% coverage [Figure 2 and Supplementary Figure S3]).

#### Co-occurrence of Viruses

**Figure 4.**
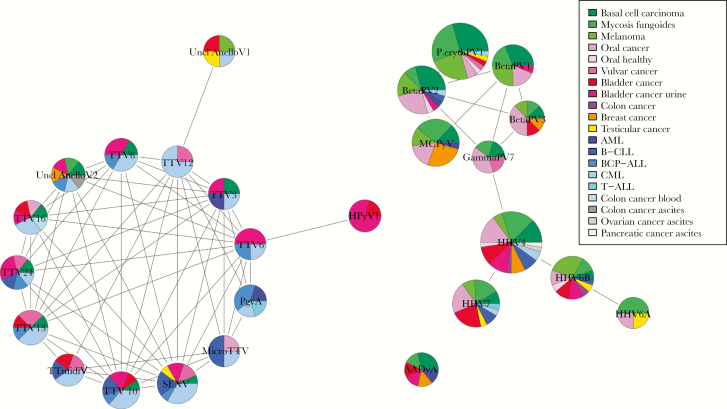
Species co-occurrence network. Network inference between the viruses grouped at species level. Nodes represent viral species, with diameters proportional to the total number of occurrences of a species (ranging from 4 to 40) and colored segments representing the proportions of sample types in which a virus occurred. Green color tones represent skin-associated sample types, red/pink color tones represent mucosal, blue represent sample types originating from blood (mainly leukemias), orange/yellow represent other tissue, and gray tones represent ascitic fluid. AADvA, adeno-associated dependoparvovirus A; AML, acute myeloid leukemia; B-CLL, B-cell chronic lymphocytic leukaemia; BCP-ALL, B-cell precursor acute lymphoblastic leukaemia; BetaPV, *Betapapillomavirus*; CML, chronic myelogenous leukemia; GammaPV, *Gammapapillomavirus*; HHV, human herpesvirus; HPyV1, human polyomavirus 1 (BKV); MCPyV, merkel cell polyomavirus; MicroTTV, micro torque teno virus; PgvA, Pegivirus A; P.erythPV1, primate erythroparvovirus 1 (parvovirus B19); SENV, SEN virus; T-ALL, T-lineage acute lymphoblastic leukaemia; TTmidiV, torque teno midi virus; TTV, torque teno virus; Uncl Anello, Unclassified Anellovirus.

The nonrandom patterns of viruses detected in the different sample types were explored by investigation of co-occurrence of viruses. For this analysis, viruses were grouped at species level, and only species identified in at least 4 samples by read mapping were included (Figure 4). Viral species clustered in 2 main groups; one mainly consisting of anelloviruses and one mainly of herpesviruses and papillomaviruses. It is interesting to note that taxonomically unrelated viruses were found to co-occur; BKV and Pegivirus A were associated with the anellovirus cluster, whereas human parvovirus and MCPyV were associated with papillomaviruses. The anellovirus cluster was associated primarily with leukemias and mucosal samples, whereas the herpes and papillomavirus cluster was associated mainly with skin-associated and mucosal sample types.

### Viruses With Nonhuman Hosts

Among the viral best BLASTnx hits for the contigs, we identified hits to viruses from 25 viral families with nonhuman hosts, as well as unclassified viruses. The majority of these “nonhuman” viruses occurred ubiquitously across sample types (Supplementary Figure S6), and detection of these seemed to be confined to the application of certain laboratory methods (Supplementary Figure S7). These are considered in the Supplementary Results and in [23].

### Evaluation of Methods Applied

Sequencing of total DNA or RNA, capture of retroviral DNA or mRNA, and mRNA enrichment showed few or no virus-positive samples. The remaining enrichment methods largely detected the same viral families, but not with the same frequency (Table 3, Supplementary Figures S8 and S9). Some of the viral findings were confirmed by more than 1 method (Supplementary Figure S10). A comparison of the methods applied in terms of number of samples positive, ability to retrieve high genome coverage, or ability to detect divergent viral sequences is presented in the Supplementary Results.

**Table 3.** Datasets Positive for a Given Viral Family for the Laboratory Methods Applied

All Samples		*Papillomaviridae*		*Polyomaviridae*		*Herpesviridae*		*Parvoviridae*		*Anelloviridae*	
Method	Datasets (n)	Contigs	Reads	Contigs	Reads	Contigs	Reads	Contigs	Reads	Contigs	Reads
Vert. virus capt. DNA	75	3	31	1	3	10	42	19	32	5	15
Circular DNA	114	5	5	4	4	1	3	6	6	13	13
Virion DNA	143	6	21	4	24	1	9		2	4	12
Virion RNA	146	1	13		1		4	1	5	6	15
Retrovirus capt. DNA	33					1	6		1		
Total DNA	107		2			1	6		3		3
Total RNA	72		1				3		2		1
Samples Processed With All 4 Methods											
Vert. virus capt. DNA	58	2	22^a^			7	30^b^	15	23^c^	4	11
Circular DNA	58	4	4			1	2	6	6	6	8
Virion DNA	58	4	14^d^	3	15		5		1	3	11
Virion RNA	58	1	12				2	1	3	5	11

Abbreviations: capt., capture; DNA, deoxyribonucleic acid; RNA, ribonucleic acid; Vert., vertebrate.

Notes: The number of datasets positive based on contig BLASTnx (leftmost column shown for each viral family) and read mapping (rightmost column shown for each viral family) are shown. The top part of the table shows the numbers for all datasets, the bottom part shows the number for datasets from samples processed with all 4 enrichment methods. Only the 5 most frequently detected families are shown, and only confirmed viral hits are included. Nontemplate controls are excluded.

^a^ *P* = 9.5 × 10^–5^ vs circular DNA enrichment.

^b^ *P* = 5.1 × 10^–7^ vs virion enrichment DNA (nonsignificant at contig level, *P* = .061).

^c^ *P* = 4.6 × 10^–4^ vs circular DNA enrichment (nonsignificant at contig level, *P* = .052).

^d^ *P* = .019 vs circular DNA enrichment.

## DISCUSSION

In the present study, we conducted a comprehensive virome investigation of 197 patient samples from 18 sample types of cancerous origin by applying a broad diversity of methods for enrichment of viral nucleic acids before sequencing. Targeting viruses with DNA and RNA genomes, double-stranded, single-stranded, and circular genomes, as well as proviruses, and encapsidated and uncoated viral nucleic acids using sensitive enrichment methods (see Supplementary Discussion), we sought to fully cover the diversity of viruses present in the cancerous material. The resulting 710 distinct metagenomic datasets were analyzed using a BLAST-based analysis approach and in-depth viral sequence analysis at both the contig and read level. Our study provides central points of awareness concerning virome data analysis that need to be addressed before interpretation of the results. This includes viral artefacts, cross-mapping between closely related species/strains, and bleedover occurring during sequencing, as well as the presence of viral sequences in nontemplate controls (see Supplementary Material).

Most of the viruses identified in our study are commonly found in humans, and they were almost exclusively DNA viruses (see Supplementary Discussion). Viral sequences were detected in a large percentage of the samples investigated, and, as expected, skin-associated and mucosal samples showed higher proportions of virus-positive samples. Only a few IARC-classified carcinogenic viruses were detected. These included the full genome of HPV16 identified in 1 of 3 vulvar cancer samples, confirming previous reports [39]. The full genome of HPV18 was detected in 1 of 10 bladder cancer urine samples. The evidence for a role of high-risk HPVs in the development of bladder cancer is currently inadequate [40, 41], and our study does not provide further support of high-risk HPVs playing a significant role. Evidence supports a causal role for HPV16 in a subset of oropharyngeal cancers [1], whereas the prevalence of HPVs in oral cavity cancer is low [42]. Therefore, the absence of high-risk HPVs is not unexpected. Read mapping suggested presence of multiple HPV types in most HPV-positive samples. However, as was seen for BKV/JCV and HHV6A/6B, it cannot be ruled out that the detection of some types occur as a result of cross-mapping between closely related types. Viruses considered possibly carcinogenic and appearing in our samples included the polyomaviruses MCPyV, BKV, and JCV. These viruses are commonly carried asymptomatically [43], and therefore the findings could represent normal flora.

A potential role for the ubiquitous anelloviruses in cancer is debated [44]. Multiple anelloviruses were often detected in the same sample, as previously reported in, for example, urine [12]; however, no specific anellovirus types recurred consistently within cancer types. At the contig level, different species or strains can more readily be evaluated and distinguished (Supplementary Table S11); however, due to the read-mapping patterns observed for some anelloviruses (see Supplementary Material) as well as possible cross-mapping, the diversity is possibly overestimated.

Parvovirus B19 was consistently detected in skin-associated samples. Seroprevalence is high in the general population, and the viral DNA can persist in multiple tissues, including skin [45, 46], although previous detection rates are lower than what was found here. Parvovirus B19 was not found in previously published skin and oral virome studies [5, 9], but these discrepancies could reflect differences in sample material and processing.

The effect of co-occurrence of viruses within a tissue is a relatively unexplored area. The co-occurrence of viral species and nonrandom distribution patterns found here reflect differences in viral tissue tropism, but other factors could play a role as well. Our study includes various habitats of the human body sampled from different individuals, providing a cross-body comparison of viral variation. Future studies of viral composition might reveal interactions of potential importance in health or disease between members of the virome.

With our study, several cancer types have been thoroughly investigated for viral nucleic acids. Cancer types investigated by us and not included in previous RNA-sequencing studies [15–17, 47, 48] include basal cell carcinoma, testicular cancer, B-cell chronic lymphocytic leukaemia (B-CLL), BCP-ALL, CML, T -ALL, vulvar cancer, and multiple myeloma cell lines. A limitation of our study is the low number of healthy control samples available, which hinders conclusions regarding viral presence in tumor versus normal flora. Although our sample size is not large, we consider the probability of uncovering yet undetected (known) viruses present in large proportions of these cancers low. Human papillomavirus 16 was detected in 1 of 3 vulvar cancer samples included, suggesting that our sample size is large enough to identify cancer-causing viruses of high prevalence. Nevertheless, low-frequency associations between known viruses and cancers might exist, and establishing causality in such cases is a complex process [49]. Other relevant approaches within cancer virus discovery include investigation of truly novel viruses with little or no similarity to known viruses, which are not detectable by the applied analysis methods. Moreover, changes in gene expression or DNA methylation may be directly induced by viral infections [50], and searching for such viral “footprints” could reveal new associations between previous viral infections and cancer.

## Supplementary Data

Supplementary materials are available at *The Journal of Infectious Diseases* online. Consisting of data provided by the authors to benefit the reader, the posted materials are not copyedited and are the sole responsibility of the authors, so questions or comments should be addressed to the corresponding author.

## Supplementary Material

jiz318_Suppl_Supplementary_Material

jiz318_Suppl_Supplementary_Figure_S6

jiz318_Suppl_Supplementary_Figure_S7

## Notes

***Acknowledgments.*** We thank Esben Nørgaard Flindt for valuable coordination of the GenomeDenmark platform. We also thank Sarah Nathalie Vitcetz for technical assistance. We furthermore thank Zoltan Szallasi (Technical University of Denmark) for establishing contact to collaborators at National Institute of Oncology, Budapest, Hungary.

***Author contributions.*** L. P. N., A. J. H., E. W., L. V., H. F., S. M., K. R. K., M. A., J. R., U. B., and S. B. conceived concept and designed the study. S. M. wrote the paper. L. V., A. J. H., M. A., H. F., K. R. K., J. M. G. I., O. L., and L. P. N. contributed significant input for editing of the manuscript. S. M., K. R. K., M. A., L. V., H. F., A. J. H., L. P. N., and T. M. contributed scientific discussions for interpretation of data. H. F., L. V., S. M., and K. R. K. developed laboratory protocols. S. M., H. F., L. V., K. R. K., S. R. R., I. B. N., C. P., A. R.-I., D. E. A.-P., P. V. S. O., and R. H. J. performed laboratory experiments. J. F.-N., J. M. G. I., O. L., T. A. H., A. J. H., T. M., S. M., S. B., and T. S.-P. developed computational pipeline or provided supervision. J. F.-N., J. M. G. I., and S. M. conducted initial bioinformatic analysis (preprocessing, assembly, BLAST, DIAMOND). S. M., M. A., K. R. K., J. F.-N., and T. M. performed further/additional/concluding analysis (data mining, investigation/confirmation of viral hits, visualization). J. A. R. H. contributed to mapping to viral genomes and creation of Circos plot. C. J. B. performed network analysis. E. R.-D. M., L. G.-P., C. v. B., D. H. J., R. G., E. H., I. P., I. V., Z. B., K. D., H. E. J., T. S., P. H., J. L. L., and J. R. conducted sample collection.

***Disclaimer.*** The funders had no role in study design, data collection and analysis, decision to publish, or preparation of the manuscript.

***Financial support.*** This study was funded by the Innovation Fund Denmark (The GenomeDenmark platform, grant number 019-2011-2), the Danish National Research Foundation (grant number DNRF94), and the Lundbeck Foundation.

***Potential conflicts of interest.*** All authors: No reported conflicts of interest. All authors have submitted the ICMJE Form for Disclosure of Potential Conflicts of Interest.

Presented in part: Third International Conference on Clinical Metagenomics, October 18–19, 2018, Geneva, Switzerland.
