# Supplementary material for: High-Throughput Sequencing-Based Investigation of Viruses in Human Cancers by Multienrichment Approach
Source: J Infect Dis. 2019 Jun 28;220(8):1312–24. doi: 10.1093/infdis/jiz318 (PMC6743825; doi:10.1093/infdis/jiz318)
Supplement: jiz318_Suppl_Supplementary_Material [file jiz318_suppl_supplementary_material.pdf]

Supplementary Material

Supplementary figures

Fig S1 can be found here:

http://www.cbs.dtu.dk/public/cancer_pathogen/circos_plots/

Username: canpath

Password: TXF7RCVn

Fig S6 and S7 are uploaded as PDFs.

Fig S1. Viral genome coverage from read mapping.

The figures show the regions to which reads were found mapping. The figures include all mapping results, i.e. both confirmed viral hits and artefacts.

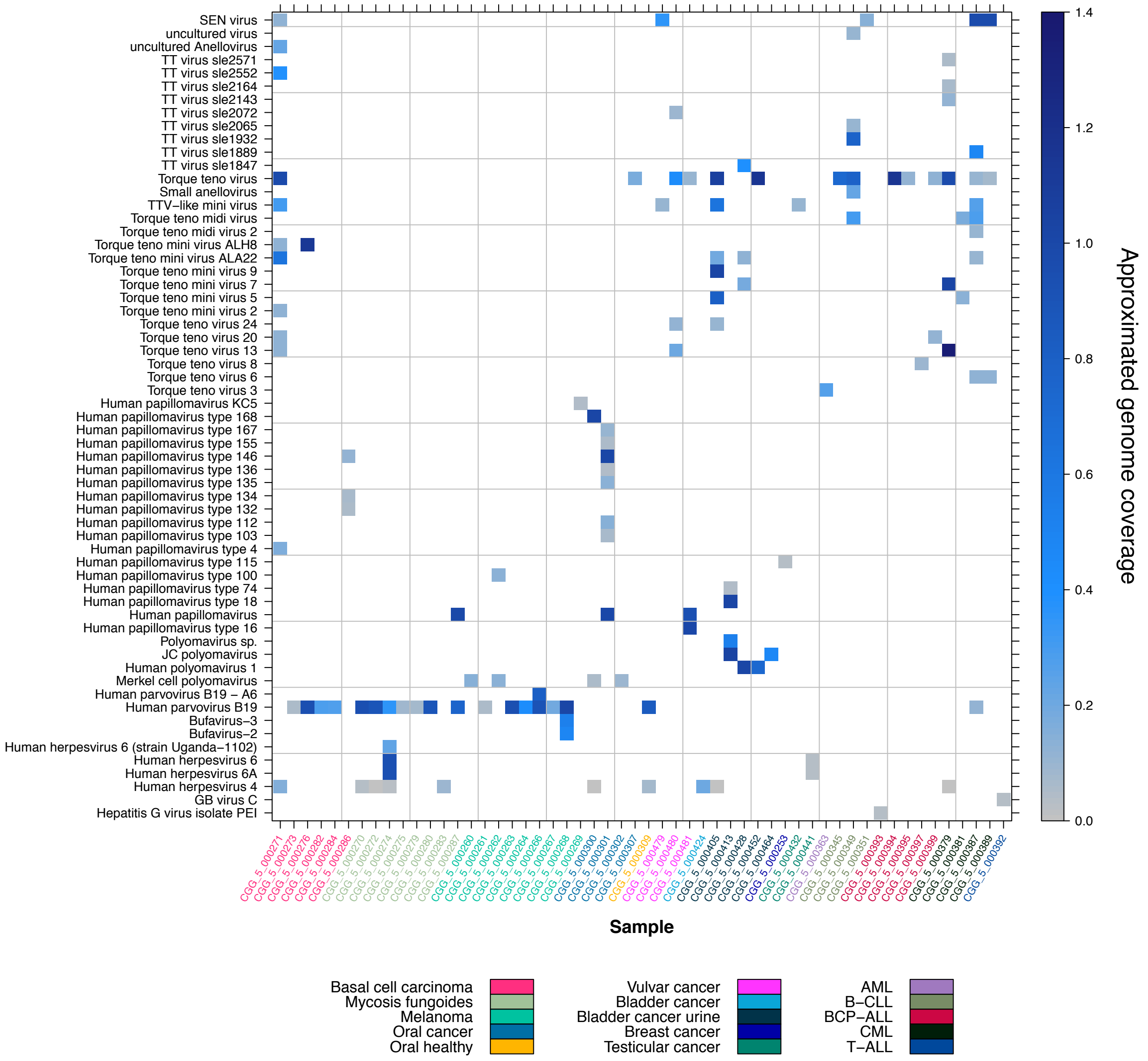

Fig S2. Viral strains detected among the contig BLASTnx alignments.

Approximated genome coverage (%) across cancer types (horizontal axis), indicated by colour (right legend). Coverage was estimated by summing up contig lengths, occasionally yielding a coverage above one, hence the values represented by the colour scale ranges from 0 to 1.4 times coverage. If a given virus was detected more than once in the same sample (i.e. with different laboratory methods), only the one having the highest coverage is shown. Due to BLAST of the contigs being run prior to the discovery of cutavirus, the cutaviral contigs identified in our data showed best BLAST hit to bufavirus-2 and 3. Only confirmed viral hits are included. NTC: Non-template control.

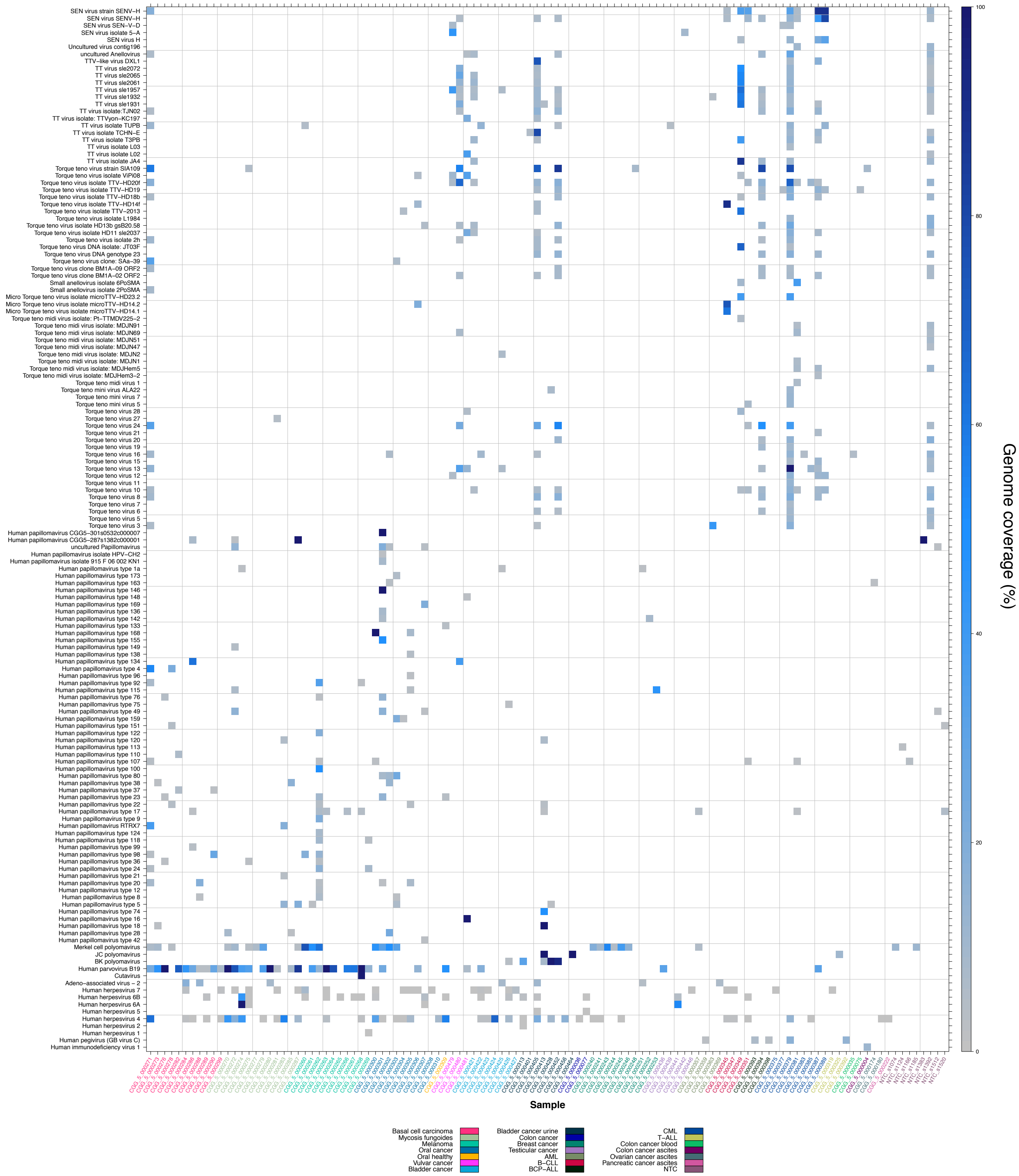

Fig S3. Viral strains detected from read mapping.

Genome coverage (%) across cancer types (horizontal axis), indicated by colour (right legend). If a given strain was detected more than once in the same sample (i.e. with different laboratory methods), only the one having the highest coverage is shown. Only confirmed viral hits are included. NTC: Non-template control.

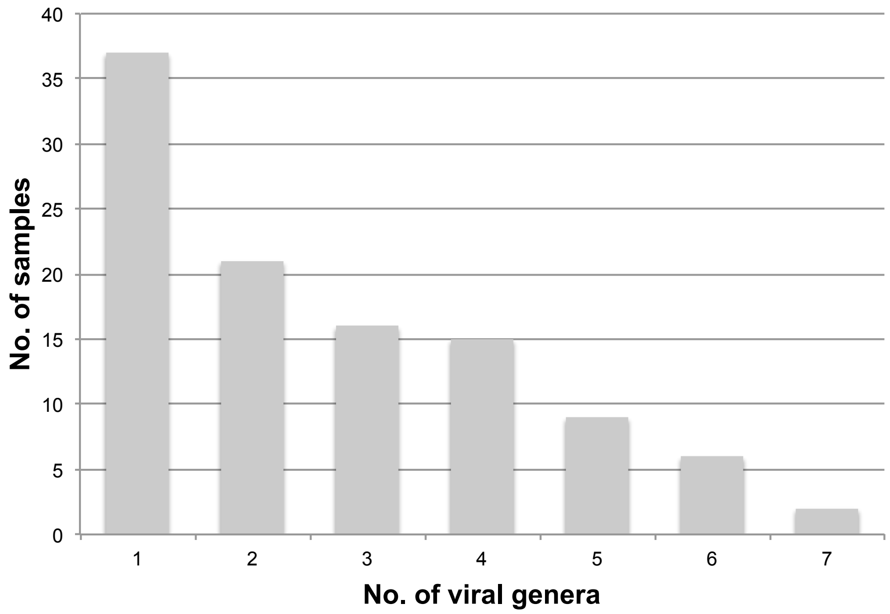

Fig S4. **Number of samples positive for a given number of viral genera based on read mapping.**

Only confirmed viral hits are included. Unclassified papillomaviruses and anelloviruses are counted as genera, despite that their members might belong to an already counted papillomavirus/anellovirus genus.

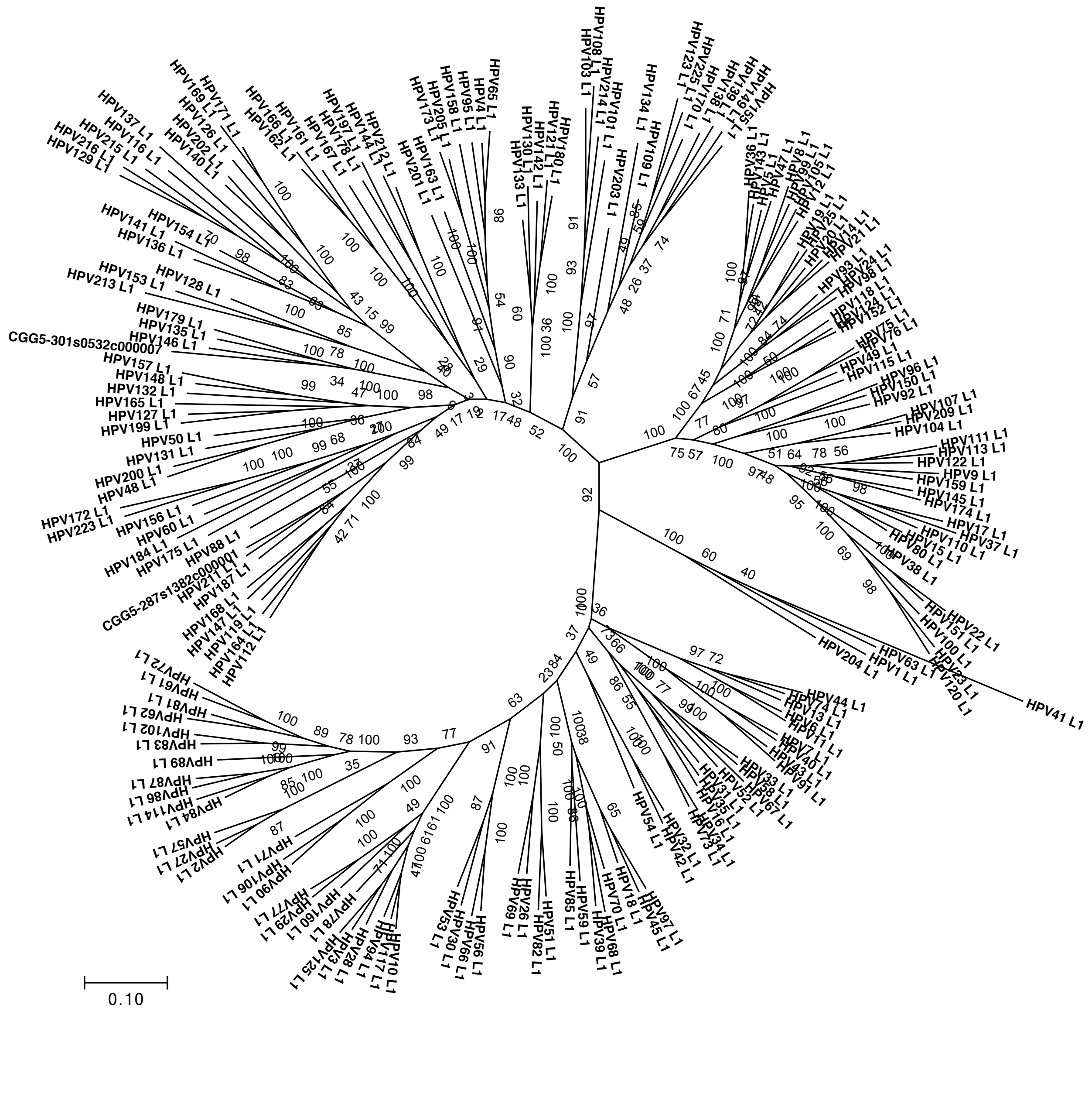

Fig. S5. Phylogenetic analysis of the novel HPVs.

Phylogenetic analysis of the two novel HPVs discovered (see Supplementary Methods below) based on L1 nucleic acid sequences. The tree was constructed by the maximum-likelihood method with 100 bootstrap replicates. The tree is drawn to scale, with branch lengths measured in the number of substitutions per site.

Fig S6. Non-human viruses detected from contig BLASTnx shown vs. cancer type.
Number of contigs detected across cancer types (horizontal axis), indicated by colour (right legend). NTC: Non-template control.

Fig S7. Non-human viruses detected from contig BLASTnx shown vs. laboratory method.
Number of contigs detected across the applied laboratory methods (horizontal axis), indicated by colour (right legend). NTC: Non-template control.

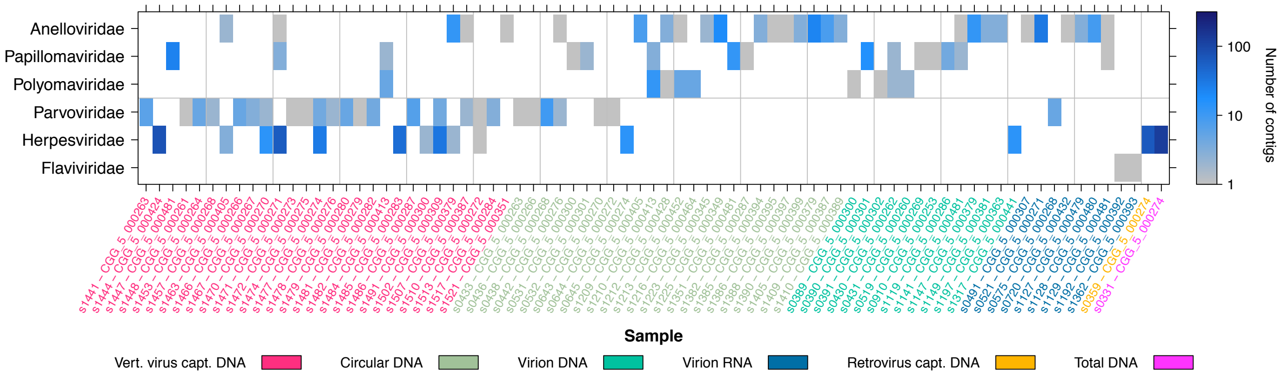

Fig S8. Viral families detected from contig BLASTnx across laboratory methods applied.
Number of contigs detected for each viral family across laboratory methods (horizontal axis), indicated by colour (right legend) Only confirmed viral hits are included. Non-template controls are excluded.

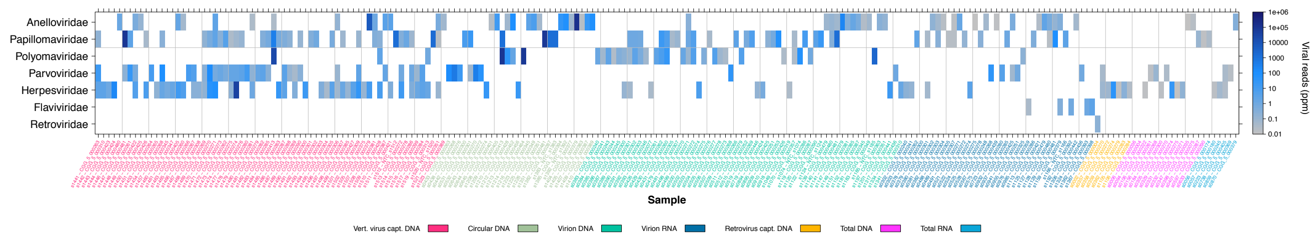

Fig S9. Viral families detected from read mapping across laboratory methods applied.
The fraction of viral reads in parts per million (ppm) for each viral family across laboratory methods (horizontal axis), indicated by colour (right legend). Only confirmed viral hits are included. Non-template controls are excluded.

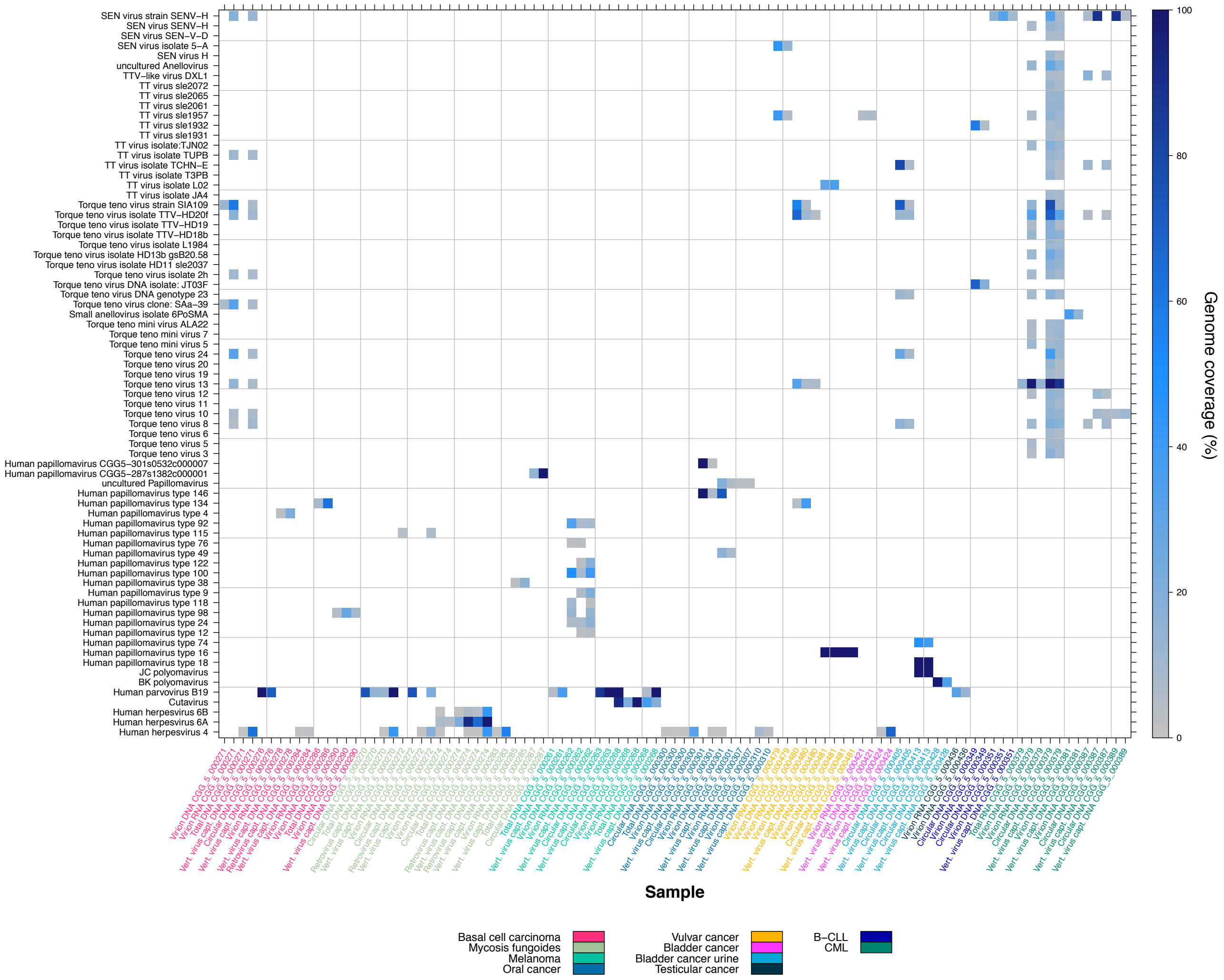

Fig S10. Viruses detected in the same sample by more than one method.

Genome coverage (in %, indicated by colour in right legend) for all occasions of viruses detected two or more times in the same sample, i.e. by different laboratory methods. The given method is shown on the x-axis next to the sample name. Only confirmed viral hits are included.

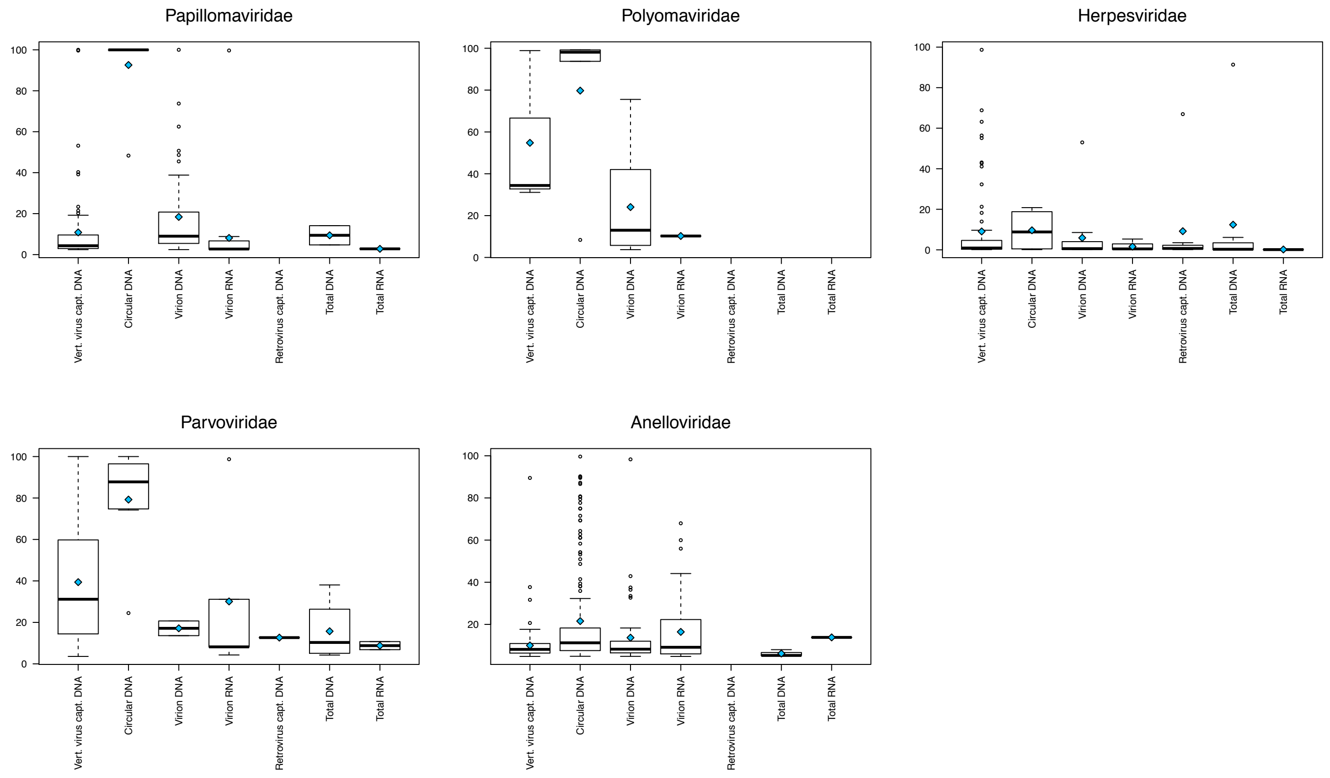

Fig S11. Genome coverage for the five main viral families detected.

The percent genome coverage based on read mapping is shown according to laboratory method for the five main viral families identified. Only confirmed viral hits are included.

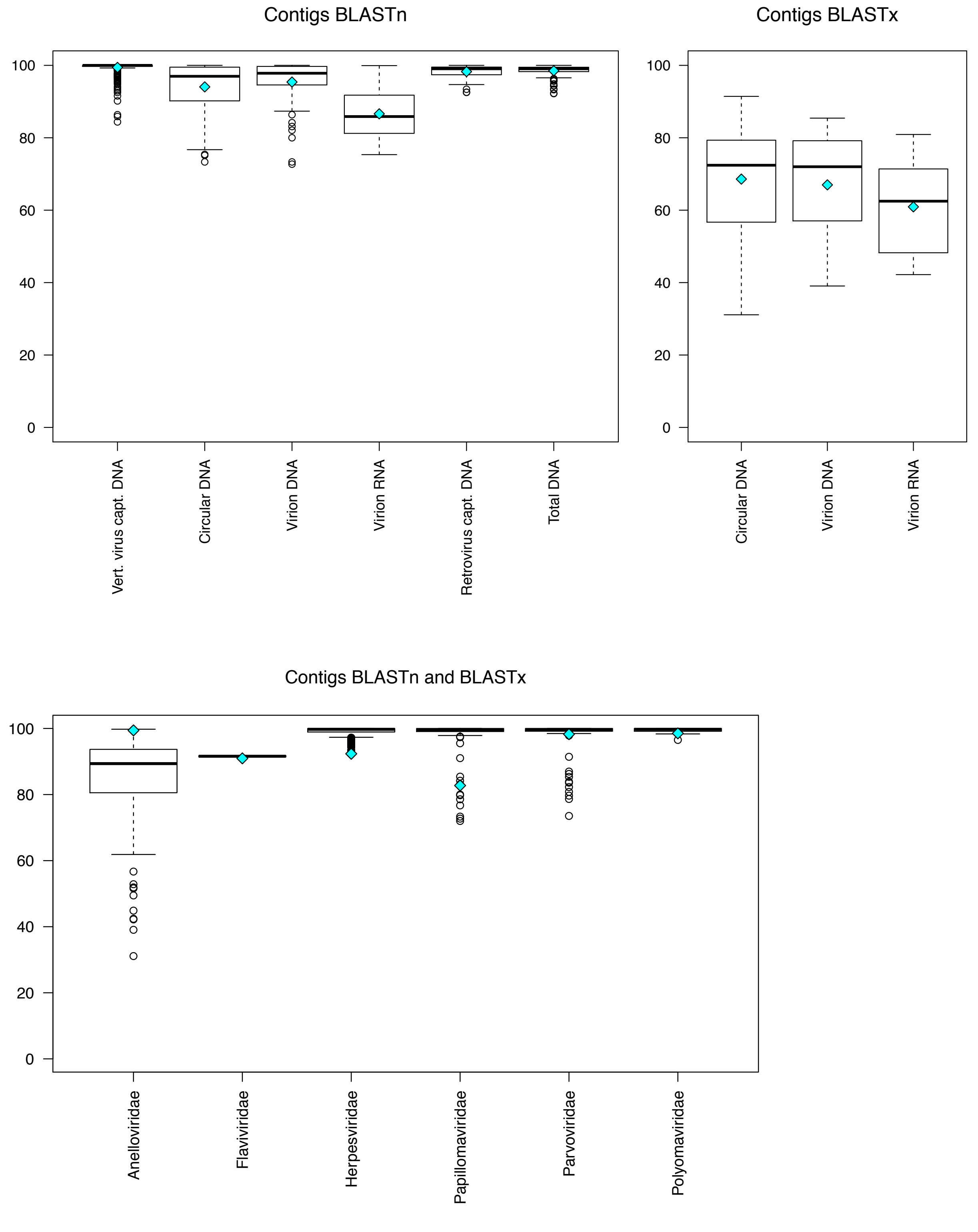

Fig S12. **Percent identity of the BLAST alignments for contigs.**

Top: The percent identity of BLASTn and BLASTx alignments according to the laboratory method applied. Bottom: The percent identity according to viral family. The black lines indicate the median, while the cyan squares indicate the mean. Only confirmed viral hits are included.

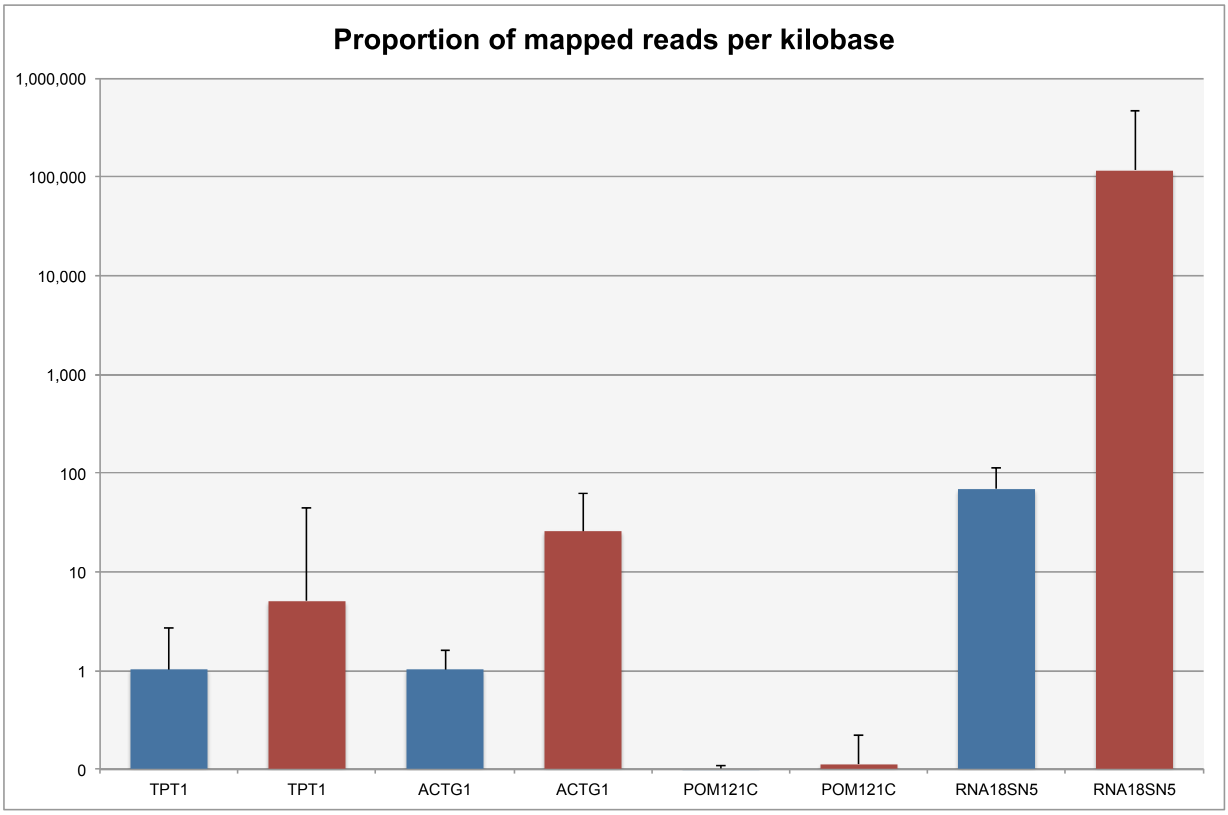

Figure S13. Mapping to RNA transcripts.

The median of the fraction of mapped reads in parts per million per kilobase (gene length) relative to the total number of reads is shown on a log scale for total DNA sequencing (blue columns) and total RNA sequencing (red columns).

TPT1: Homo sapiens tumor protein, translationally-controlled 1; ACTG1: Homo sapiens actin gamma 1; POM121C: Homo sapiens POM121 transmembrane nucleoporin C; RNA18SN5: Homo sapiens RNA, 18S ribosomal N5.

Supplementary tables

Table S1, S2, S4, S5, S6, S8, S10, S11, and S14 can be found here:

http://www.cbs.dtu.dk/public/cancer_pathogen/supplementary_data/

Username: canpath

Password: TXF7RCVn

Table S1. Information about samples and datasets.

List of the individual samples included in the study, including cancer type, method applied, read counts, contig counts and viral hit counts.

Table S2. Viral genomes included in capture assays.

Viral genomes included in retrovirus capture v1, retrovirus capture v2, and vertebrate virus capture are shown.

Table S3. Read and contig counts.

Method	Datasets (n)	Median no. of demultiplexed reads	Median no. of non-human reads	No. datasets having contig BLAST results	Median contig count
Vert. virus capt. DNA	75	30,567,831	2,838,773	75	657
Circular DNA	114	48,624,897	205,990	114	227
Virion DNA	143	53,893,043	680,286	143	145
Virion RNA	146	50,754,731	2,535,555	146	147
Retrovirus capt. DNA	33	49,821,266	300,076	33	301
Retrovirus capt. mRNA	6	30,501,521	651,990	6	38
Total DNA	107	104,899,499	293,813	105	658
Total RNA	72	76,230,360	1,025,115	67	1149
mRNA	14	168,693,407	2,537,740	14	2548
Total	710	57,197,561,075	3,121,807,453	695	1,455,489

Median number of demultiplexed and human-depleted read pairs, number of datasets having contig BLASTnx results, and median contig count. The bottom row shows the total read or contig counts. Non-template controls are excluded from the counts.

Table S4. All human viruses identified by BLAST.

Human viruses detected from BLASTnx of contigs and BLASTn of reads, and list of confirmed human viruses and human virus artefacts from BLASTnx of contigs.

Table S5. Viral references included in the read mapping analysis.

Lists of references to which no reads map, reference with coverage below the cut-off only, references showing confirmed viral hits, and viral artefacts are also included. Furthermore, anellovirus references are classified as showing either full/dispersed coverage or primarily showing mapping in the untranslated region.

Table S6. Read count and coverage from read mapping.

The min, median, and max of read count, coverage in percentage, and coverage in bp for mapping to the viral reference genomes are shown for all mappings, confirmed viral hits, and viral artefacts.

Table S7. Number and percentage of virus positive samples.

Cancer	Samples (n)	Contig BLASTnx		Read mapping	
		No. positive	% positive	No. positive	% positive
Basal cell carcinoma	11	6	55	11	100
Mycosis fungoides	11	8	73	11	100
Melanoma	10	9	90	10	100
Oral cancer	10	4	40	10	100
Oral healthy	1	1	100	1	100
Vulvar cancer	3	3	100	3	100
Bladder cancer	7	1	14	7	100
Bladder cancer urine	10	5	50	8	80
Colon cancer	16	0	0	2	13
Colon healthy	2	0	0	0	0
Breast cancer	20	1	5	10	50
Testicular cancer	20	2	10	5	25
AML	9	1	11	4	44
B-CLL	9	3	33	4	44
BCP-ALL	8	5	63	3	38
CML	10	4	40	8	80
T-ALL	11	1	9	3	27
DLBCL	5	0	0	0	0
Lymphoblastic lymphoma	1	0	0	0	0
Multiple myeloma	6	0	0	0	0
Colon cancer blood	8	0	0	2	25
Colon cancer ascites	1	0	0	1	100
Breast cancer ascites	1	0	0	0	0
Ovarian cancer ascites	5	0	0	2	40
Pancreatic cancer ascites	2	0	0	1	50
NTC	50	0	0	8	16
Total (without NTCs)	197	54		106	

The number of samples, number of samples blasted, and number and percentage of virus-positive samples based on BLASTnx of contigs and read mapping. Only confirmed viral hits are included. NTC: Non-template control.

Table S8. Number of samples positive for a given virus.

The number of samples showing detection of a given virus based on BLASTnx of contigs (left) or read mappings (right) are shown. Only confirmed viral hits are included.

Table S9. Viral diversity according to cancer type.

Cancer	Samples (n)	Contig BLASTnx		Read mapping	
		Families	Genera	Families	Genera
Basal cell carcinoma	11	4	6	5	11
Mycosis fungoides	11	3	4	5	12
Melanoma	10	3	5	5	8
Oral cancer	10	4	5	5	10
Oral healthy	1	2	2	3	5
Vulvar cancer	3	2	3	3	5
Bladder cancer	7	1	1	5	9
Bladder cancer urine	10	4	6	5	11
Colon cancer	16	0	0	1	3
Breast cancer	20	1	1	5	7
Testicular cancer	20	2	2	3	3
AML	9	1	1	5	6
B-CLL	9	1	1	3	6
BCP-ALL	8	2	3	2	3
CML	10	3	6	5	9
T-ALL	11	1	1	3	3
Colon cancer blood	8	0	0	1	2
Colon cancer ascites	1	0	0	2	2
Ovarian cancer ascites	5	0	0	2	2
Pancreatic cancer ascites	2	0	0	1	1
NTC	50	0	0	3	5

Number of viral families and genera detected in the different cancer types based on BLASTnx of the contigs and read mapping. Only confirmed viral hits are included. NTC: Non-template control. Unclassified papillomaviruses and anelloviruses are counted as genera, despite that their members might belong to an already counted papillomavirus/anellovirus genus.

Table S10. All HPV contigs identified.

All HPV contigs were manually re-blasted using NCBI web-based BLASTn (blastn algorithm) and mapped to the closest reference genomes using Geneious software to ensure correct assignment. The table shows details about the nearest relative, percent coverage of the reference, percent identity (id) relative to the reference, percent id in the L1 region of novel HPV sequences (if present), and number of contigs identified. The HPV hit initially identified by BLAST is also shown for comparison. Accession numbers are included for sequences uploaded to GenBank.

Table S11. TTV contigs > 1000 nt.

All TTV contigs longer than 1000 nt were manually re-blasted using NCBI web-based BLASTn (blastn algorithm) and mapped to the closest reference genomes using Geneious software to ensure correct assignment. The table shows details about the nearest relative, percent coverage of the reference, and percent identity (id) relative to the reference. The best hit initially identified by BLAST is also shown for comparison.

Table S12. Number and percentage of virus positive samples according to laboratory method applied.

All samples		Contig BLASTnx			Read mapping	
**Method**	**Datasets (n)**	**No. blasted**	**No. positive**	**% positive**	**No. positive**	**% positive**
Vert. virus capt. DNA	75	75	28	37	64	85
Circular DNA	114	114	25	22	26	23
Virion DNA	143	142	13	9	55	38
Virion RNA	146	139	9	6	35	24
Retrovirus capt. DNA	33	33	1	3	7	21
Total DNA	107	105	1	1	14	13
Total RNA	72	67	0	0	6	8
Retrovirus capt. mRNA	6	6	0	0	0	0
mRNA	14	14	0	0	0	0

Samples processed with all four methods		Contig BLASTnx			Read mapping	
**Method**	**Datasets (n)**	**No. blasted**	**No. positive**	**% positive**	**No. positive**	**% positive**
Vert. virus capt. DNA	58	58	21[a]	36	47[b]	81
Circular DNA	58	58	16	28	18[c]	31
Virion DNA	58	58	8	14	35[d]	60
Virion RNA	58	58	6	10	21[e]	36

The number of samples, number of samples blasted, and number and percentage of virus-positive samples based on BLASTnx of contigs and read mapping. The top part of the table shows the numbers for all datasets, the bottom part of the table shows the numbers for datasets from samples processed with all four included enrichment methods. Only confirmed viral hits are included. Non-template controls are excluded.

[a] P=0.0094 vs. virion enrichment DNA and non significant vs. circular DNA enrichment (P=0.43), [b] P=0.024 vs. virion enrichment DNA and $P=1.6*10^{-6}$ vs. contig BLAST, [c] non significant vs. contig BLASTnx (P=0.84), [d] $P=3*10^{-7}$ vs. contig BLASTnx, [e] P=0.0018 vs. contig BLASTnx.

Table S13. Bleedover among viral hits for the read mappings.

Method	Index	Total no. of viral hits	No. of hits ascribed to bleedover	Pct. bleedover cases	Total no. of confirmed hits ≥threshold*	No. of confirmed hits ≥threshold causing bleedover	Pct. of confirmed hits ≥threshold causing bleedover	Median viral read count for all hits ≥threshold
Vert. virus capt. DNA	Double	323	17	5	6	3	50	781525
Circular DNA	Single	441	222	50	83	42	51	1909
Virion DNA	Single	149	12	8	26	6	23	728
Virion RNA	Single	98	5	5	6	2	33	464
Retrovirus capt. DNA	Single	14	4	29	1	1	100	5311
Total DNA	Single	22	5	23	1	1	100	4541

The table shows the total number of viral hits, the number of hits ascribed to bleedover, the percentage of bleedover cases, the number of viral hits with read count above the bleedover causing threshold, the number and percentage of hits causing bleedover, and the median read count for hits above the bleedover causing threshold. The number of samples in our dataset which theoretically could give rise to bleedover was assessed based on the notion that the occurrence of bleedover requires a certain number of viral reads (≥200 for single indexing; ≥40,000 for double indexing). Comparison to the number of samples actually giving rise to bleedover shows that from 23-100% of the theoretical bleedover-causing samples actually were found to give rise to bleedover. For vertebrate virus capture, for which double indexing was used, fewer cases of bleedover were observed than could be expected based on the magnitude of the viral read count, confirming that double indexing reduces the extent of bleedover substantially. The numbers for retrovirus capture DNA and total DNA sequencing are included in the table, however, the fraction of bleedover cases for these methods are based on very low numbers of viral hits. *200 reads for single indexed libraries; 40,000 for double indexed libraries.

Supplementary Data

Supplementary Data 1 and 2 can be found here:

http://www.cbs.dtu.dk/public/cancer_pathogen/supplementary_data/

Username: canpath

Password: TXF7RCVn

Supplementary Data 1. All read mapping results.

Results of mapping to the 343 viral references. Supplementary Data 1 includes all mapping results.

Supplementary Data 2. Read mapping results for confirmed viral hits.

Mapping results for the confirmed viral hits showing coverage above 180 bp and depleted of hits arising from bleedover.

Supplementary Methods

Previously published datasets

A previous study describing a sequence clustering method [1] includes 643 of the datasets (Table S14). This study also describes the initial bioinformatic analysis (pre-processing, human depletion, *de novo* assembly, and BLAST) performed for datasets s0001-s1411 of the present study. A smaller subset of 64 datasets (from 33 samples) were included in another study concerning the retrovirus capture v1 probe panel applied here [2], and 2 datasets in a study describing the finding of cutavirus in a sample of malignant melanoma [3].

Patient samples

Superscripts below indicate sampling locations: [1]Bispebjerg Hospital, Copenhagen, Denmark; [2]Danish CancerBiobank, Herlev Hospital, University of Copenhagen, Denmark; [3]Aarhus University Hospital, Aarhus, Denmark; [4]Rigshospitalet, Copenhagen, Denmark; [5]Aalborg University Hospital, Aalborg, Denmark; [6]National Institute of Oncology, Budapest, Hungary.

The samples included in the study were: Biopsies of cutaneous basal cell carcinoma[1] (BCC) (n=11), bladder urothelial carcinoma[2] (n=7), breast cancer[2] (ductal (n=10) or lobular (n=10) carcinoma), colon cancer[2] (adenocarcinoma (n=8), mucinous adenocarcinoma (n=2), signet ring cell carcinoma (n=2), unspecified (n=4)), cutaneous malignant melanoma[3] (n=10), mycosis fungoides[1] (cutaneous T-cell lymphoma) (n=11), head and neck squamous cell carcinoma[4] (oral cavity) (n=10), testicular cancer[4] (all samples from germ cell tumours: seminoma (n=11), non-seminoma (n=5), or a mix of the two (n=4)), and vulvar squamous cell carcinoma[7] (n=3). Cells sorted by fluorescence activated cell sorting (FACS) of acute myeloid leukaemia[3] (AML) (n=9), B-cell chronic lymphocytic leukaemia[3] (B-CLL) (n=9), and chronic myelogenous leukaemia[3] (CML) (n=10); FACS sorted cells[3] (n=9) or bone marrow[4] (n=2) of T-lineage acute lymphoblastic leukaemia (T-ALL), and bone marrow of B-cell precursor acute lymphoblastic leukaemia[4] (BCP-ALL) (n=8); cryopreserved, fully

transformed cell lines[5] of diffuse large B-cell lymphoma (DLBCL) (n=5), multiple myeloma (n=6), and lymphoblastic lymphoma (n=1); blood samples[2] and healthy colon tissue from abovementioned colorectal cancer patients (n=10), urine samples[2] from bladder cancer patients, and ascites from breast[2] (n=1), colorectal[2] (n=1), ovarian[2] (n=5), and pancreatic[2] (n=2) cancer patients. Ascites samples were subjected to low speed centrifugation upon reception to pellet intact host cells and cellular debris. Pelleted cells were used for shotgun sequencing, whereas supernatant was used for virion enrichment. Samples were stored in either RNAlater, Hank's Balanced Salt Solution, PAXgene, or cryopreserved in cell culture medium. All samples are listed in Table S1.

Total RNA and mRNA analysis extraction kits, library preparation and use of rRNA depletion

Total RNA analysis: High Pure Viral RNA kit (Roche) was used for colon cancer biopsies, bladder cancer urine, breast, ovarian, and pancreatic cancer ascites. RNeasy Mini Kit (Qiagen) was used for the leukaemias. QIAamp DNA Mini Kit (Qiagen) was used for breast cancer biopsies.

mRNA analysis: extraction was performed using Dynabeads mRNA Direct Purification Kit (Invitrogen).

Library preparation: ScriptSeq v2 RNA-seq Library Preparation Kit (Epicentre) was used for all samples subjected to total RNA analysis excluding colon cancer biopsies and ovarian cancer ascites. ScriptSeq Complete Gold Library Preparation Kit, which includes rRNA depletion, was used for colon cancer biopsies and ovarian cancer ascites subjected to total RNA analysis and for all samples subjected to mRNA analysis, including retrovirus mRNA capture. Only mRNA libraries were stranded due to the preceding for polyA-selection.

Sequencing and data analysis

Pre-processing, human depletion, assembly, and taxonomical classification: Paired-end sequencing (2×100 bp) was performed on the Illumina HiSeq 2000 platform at BGI-Europe (Copenhagen N, Denmark). The datasets were analysed in two batches, with slight analytical differences. The

analysis of datasets no. s0001-s1411 (Table S1) is detailed in [1]. Briefly, paired-end sequencing reads were trimmed of adapter sequences and overlapping read pairs merged using AdapterRemoval [4] (version 1.5.3). Reads shorter than 30 nucleotides after trimming were excluded from further analysis. Depletion of human sequences was performed by mapping the reads to the human genome (hg38, including decoys and alternative sequences*) using BWA [5] (version 0.7.7). Reads of a pair were evaluated independently, and unmapped reads were kept for further analysis. Reads containing 25 bp or more of low complexity regions were filtered out using the DustMasker algorithm[6] (version 1.0.0). *De novo* assembly of the reads into contigs was achieved using IDBA[7] (version 1.1.1) with default parameters. For taxonomic classification, the reads and contigs were aligned to the NCBI nucleotide database (*nt*) using BLASTn (megablast) [8] with a cut-off e-value of 10^{-3}. The best hit was defined as the alignment with the highest bit-score. Regions in the contigs having BLASTn hits were masked, and the contigs were aligned against the NCBI non-redundant protein database (*nr*) using BLASTx with a cut-off e-value of 10^{-3}.

For the remaining datasets (s1431-s1523), the following differences in analysis apply: depletion of human reads was performed using Bowtie2 [9] (version 2.0.0) with end-to-end alignment to the human genome (hg19). No lower cut-off for read length was applied. Contigs with no BLASTn hit were aligned to the NCBI *nr* database using DIAMOND[10] instead of BLASTx. Reads were not blasted.

*The human genome version hg38 with decoys includes the genome of human herpesvirus 4 (chrEBV), and reads mapping to this virus were consequently removed during depletion of human reads. Reads mapping to chrEBV were therefore extracted from the bam files and re-mapped to HHV4 (NC_007605) using Bowtie2 [9] (version 2.2.9) with end-to-end alignment and exclusion of reads shorter than 50 nt. The results were included in the results from mapping to the viral reference genomes and processed along with these (see below).

Investigation of human viral hits

To exclude false positives among the viral hits, all hits to human viruses were evaluated *in silico* and categorised either as confirmed viral hits or artefacts. For the contigs, the BLAST/DIAMOND hits were evaluated manually by aligning the contigs to the appropriate reference genomes using Geneious software (version 7.1.7) and/or by NCBI web-based re-blast. All hits to human papillomaviruses and all anellovirus contigs >1000 nt were manually assigned at strain level based on the full length of the contig (Table S10 and S11), as these were often incorrectly assigned by BLAST in cases of low sequence identity.

For investigation of the read BLASTn hits, we compiled a database of 343 viral reference genomes representing the species/strains found with either contig or read BLAST, and for which full length sequences were available. One representative was chosen for each species/strain, and for taxa representing several different and less related strains having low sequence similarity (e.g. taxID 10566 "Human papillomavirus" and taxID 68887 "Torque teno virus"), references were chosen to represent specific strains identified by BLAST. Two novel human papillomavirus genomes identified from the contigs (HPV strain CGG5-287s1382c000001 and strain CGG5-301s0532c000007, GenBank accession numbers MG869604 and MG869605), and a cutavirus genome previously identified in one of the malignant melanoma samples [3] were also included in the database. While this article was finalised, an HPV genome with an overall identity of 97% to HPV strain CGG5-287s1382c000001 was published in GenBank, and this strain consequently no longer represents a novel HPV type. Evaluation of the read BLASTn hits was done by aligning reads of 50 nt or longer to the compiled reference database using Bowtie2 [9] with default global end-to-end alignment and reporting of the best alignment for each read. The alignment results were visualised using Circos [11]. All plots were visually inspected. If the mapped reads were distributed across the genome, the virus in question was considered a confirmed viral hit. In cases of no reads mapping or reads mapping in a regionally repeated pattern, the identification of the virus in question was considered an artefact.

Bleedover, or miss-assignment of reads to the wrong index sequence, is known to occur between libraries sequenced on the same lane of the flow cell [12],[13]. If reads mapping to the same virus were detected in more than one dataset sequenced on the same lane, read count ratios were calculated relative to the dataset with the highest read count. A cut-off of 0.5% (0.5%x0.5% for double indexed libraries) was applied and hits below this cut-off were disregarded as bleedover artefacts. This threshold is higher than the level previously reported [12] and was empirically determined based on our data, as hits suspected of being bleedover often presented themselves at read count ratios just below 0.5%. Furthermore, all contigs from datasets sequenced on the same lane and having hits to the same virus were compared. In cases of inter-sample high identity matches, the read counts and coverage patterns from mapping to the virus in question were taken into account, and contigs originating from datasets having read counts below the bleedover cut-off were also categorised as bleedover artefacts.

For the read mapping, a lower cut-off of 180 bases covered was applied to reflect a minimum of two reads mapping to a given virus in separate positions. For human immunodeficiency virus a cut-off of 205 nt was applied, as reads mapping in a repetitive pattern at a coverage of 50-204 nt believed to be an artefact were seen in 85 datasets (Table S6). The results were processed using the software *R* [14] and visualised using the lattice package [15].

Phylogenetic analysis

Phylogenetic analysis of the two novel HPVs described above was performed by aligning the nucleic acid sequences of the L1 genes for these and the HPVs found in the papillomavirus genome database (PaVE). The sequences were aligned using MUSCLE (https://www.ebi.ac.uk/Tools/msa/muscle/). The phylogenetic tree was built using the Maximum Likelihood method based on the Tamura-Nei model with 100 bootstrap replicates. The tree was visualized using MEGA7 software [16].

Mapping to RNA transcripts

Based on a meta-analysis of expression profile datasets [17], three transcripts proposed as references across human tissues were chosen for the mapping analysis. These were: Homo sapiens tumor protein, translationally-controlled 1 (TPT1) transcript variant 1, mRNA (accession no. NM_001286272.1); Homo sapiens actin gamma 1 (ACTG1), transcript variant 1, mRNA (accession no. NM_001199954.2); and Homo sapiens POM121 transmembrane nucleoporin C (POM121C), transcript variant 1, mRNA (accession no. NM_001099415.3). In addition, a ribosomal RNA sequence was included (Homo sapiens RNA, 18S ribosomal N5 (RNA18SN5), ribosomal RNA, accession no. NR_003286.4).

Sequencing reads (before depletion of human sequences) from total RNA sequencing datasets, and total DNA sequencing datasets (for comparison), were mapped to the abovementioned references using Bowtie2 [9] with end-to-end alignment and the --no-mixed option to exclude reads that do not map concordantly or discordantly.

Supplementary Results

Investigation of human viral hits

BLASTnx or BLASTn of the contigs and reads identified sequences representing 74 and 349 viral species or strains infecting humans, respectively (Table S4). Further investigation of these BLAST hits revealed artefacts arising mainly due to short local-only sequence similarity of contigs or reads to viral genomes. All contig hits to human viruses were therefore evaluated manually *in silico* (see Supplementary Methods). This analysis yielded confirmed hits to 61 viruses from 6 viral families, while 14 human viruses were disregarded as false positives (Table S4) (for Bufavirus-3 (now cutavirus), both confirmed hits and artefacts were found). Overall, 9.7% of all the viral hits for contigs were confirmed as being human viral hits. Due to the large number of human viruses

detected from BLASTn of the reads, confirmation or rejection of these was achieved by mapping the reads to a database of 343 manually selected viral genomes (see Supplementary Methods), referred to as read mapping (full set of mapping results is provided in Supplementary Data 1). Visual inspection of the generated coverage plots (Fig S1) confirmed viral hits to 146 reference genomes, while the remaining either showed no reads mapping, only showed coverage below the applied cut-off, or were classified as artefacts (Table S5, and see below). For HHV1, HHV5, and HIV-1 both confirmed viral hits and artefacts were observed in separate datasets. Only *in silico* verification of viral hits was performed, except for Merkel cell polyomavirus (MCPyV), which was investigated further by PCR (see Supplementary Discussion).

Viral artefacts

From the results of read mapping, no reads were found to map to 138 of the 343 viral genomes included (Table S5). This result suggests that the identification of these viral sequences by read BLASTn is an artefact, which in many cases was found to occur due to local (involving only a shorter part of the read) sequence similarity. The results from mapping to 45 of the viral genomes were excluded due to coverage only occurring below the 180 bp coverage cut-off. For 17 of the included references, mapping artefacts were identified. For 13 of these, reads mapped in regionally repetitive patterns across datasets (Figure S13). These artefacts include human adenovirus C, hepatitis C virus, human T-lymphotropic virus 1, influenza A virus, HHV1 and 5, and human immunodeficiency virus 1 (HIV-1) (Table S5), some of which have been highlighted in previous publications [18],[19],[20]. For example, we found reads mapping to human adenovirus C (from 1-2708 reads, 0.14-13.4% coverage) (Table S6) primarily in three regions of the viral genome (Figure S12), which according to manual re-blast showed homology to expression vectors. This was particularly evident in datasets originating from samples subjected to vertebrate virus capture, where shorter adenovirus contigs also were detected, leading to classification of adenovirus as an artefact at both read and contig level. Similarly, we detected from 1-160 reads (53-116 nt coverage)

with hits to influenza A virus segment 7 (Table S6). A BLAST search of the regions of the influenza virus reference to which the reads mapped, revealed 100% identity to a vector sequence, strongly suggesting that detection of influenza A virus in our data is an artefact.

In datasets from virion enrichment RNA, reads mapping to adeno-associated virus occurred only in the region encoding the X gene, which could indicate that this finding is an artefact. However, as reads mapping to other regions of the genome occurred in datasets from other methods, adeno-associated virus was kept as a confirmed viral finding.

For some anelloviruses, reads mapped only in the untranslated region of the genome, and consequently also followed a repetitive pattern. As anelloviruses are highly divergent and the untranslated region is the least variable region of the genome, we do not consider this phenomenon an artefact as such, however, detection of reads mapping in this region only, is likely indicative of a related anellovirus strain being present in the sample, rather than the strain identified by BLAST and subsequent mapping. All anelloviruses were therefore categorised as showing either dispersed to full coverage or only showing coverage in the conserved region (Table S5). The latter category likely represents detection of related viruses not included in the mapping database.

Viruses with non-human hosts

The non-human viruses included hits likely arising as a result of laboratory component-derived contamination, an issue which we have explored in detail elsewhere [21] (article in press). To search for potential novel viruses more distantly related to human viruses, several non-human viral contigs were investigated further. Among others, these included several parvoviruses (e.g. Muscovy duck parvovirus, Tumor virus X, mouse/rat/bovine parvoviruses) and a herpesvirus (Murid herpesvirus). In most cases we identified only a single short contig (2-300 nt) in a given sample. Manual re-blast revealed either non-viral hits or short fragmented stretches of sequence identity to viruses. Many of the contigs were detected in leukaemia samples or lymphoma cell lines, which often had been stored in cell culture medium. Some of these viral hits (e.g. bovine viruses) could

therefore originate from calf serum added to the culture medium. Consequently, no credible novel human viral sequences were identified among the non-human viral sequences.

Evaluation of methods applied

Nine different methods were used for sample processing (Fig 1, Table 1). Sequencing of total DNA or RNA, capture of retroviral DNA or mRNA, and mRNA enrichment showed few or no virus-positive samples, and were not included in the following comparison. To compare the performance of the four remaining enrichment methods (vertebrate virus capture, virion enrichment DNA and RNA, and circular DNA enrichment) we compared the subset of samples processed with all four methods (n=58, >29% of the included samples) (Table S12). Vertebrate virus capture showed the highest number of confirmed virus-positive samples. The difference was significant compared to virion enrichment DNA ($P < 0.01$ for contig BLASTnx (21 vs. 8 samples), $P < 0.05$ for read mapping (47 vs. 35 samples)), which showed the third and second highest number of positive samples for contigs and reads, respectively. Read mapping identified significantly higher numbers of virus-positive samples than contig BLASTnx ($P < 0.01$), except for circular DNA enrichment. The four methods largely detected the same viral families, but not with the same frequency (Table 3, Fig S8-S9). Anelloviruses were found in comparable numbers of datasets for all four methods. Herpesviruses and parvoviruses were found in the highest number of datasets using vertebrate virus capture ($P < 0.001$ for read mapping). For papillomaviruses, vertebrate virus capture and virion enrichment DNA showed significantly higher numbers compared to circular DNA enrichment at read mapping level ($P < 0.001$ and $P < 0.05$, respectively). Circular DNA enrichment retrieved higher viral genome coverage than the other methods, especially for papillomaviruses, polyomaviruses, and parvoviruses (Fig S11). The more divergent viral contigs were found when applying circular DNA enrichment and virion enrichment DNA and RNA (Fig S12), identifying BLASTnx alignments with percent nucleotide identity as low as 31-42%. In contrast, the lowest identity BLASTnx hit found when applying vertebrate virus capture showed 84% identity to the

reference. The lowest identity hits were generally detected by BLASTx as expected, and mainly represented anelloviruses. Of the contigs representing confirmed viral hits, 962 were assigned to viruses by BLASTn, while an additional 46 contigs were assigned by BLASTx.

More than 200 viral hits were ascribed to suspected bleedover in samples processed by circular DNA enrichment, while fewer than 20 bleedover cases were found for each of the remaining enrichment methods (Table S13). For vertebrate virus capture, having double indexed sequencing libraries, fewer cases of bleedover were observed than could be expected, confirming that double indexing reduces the extent of bleedover substantially, as previously shown [12].

Verification of RNA-sequencing

To verify if our RNA-sequencing methodology in fact did target RNA templates, we performed mapping of sequencing reads to selected RNA transcripts and ribosomal RNA. Mapping was only performed for total RNA sequencing datasets (and total DNA sequencing for comparison), as the virion enrichment RNA method involves depletion of human sequences by nuclease treatment. The fraction of reads mapping in parts per million per kilobase (gene length) relative to the total number of reads was calculated (Figure S13). The analysis revealed a median ppm value 4.9 times higher for RNA than DNA for the TPT1 transcript, 25 times higher for the ACTG1 transcript, 1.6 times higher for the POM121C transcript, and >1600 times higher for the ribosomal RNA sequence.

Supplementary Discussion

Sensitivity and limitations of analysis methods

The sensitivity of viral sequence enrichment methods is nowadays comparable to sequence-specific PCR detection, which in contrast is limited by its specificity for specific sequences, and therefore

may fail detecting divergent viral sequences. The high sensitivity of several of the applied methods compared to shotgun sequencing of non-enriched material was demonstrated in previous studies [2],[22]. There are limitations to some of these methods, for instance in assuming that viral particles remain intact during the enrichment procedure prior to extraction, or that RNA is not degraded.
The BLAST-based analysis approach was chosen due to, at the time, lack of comprehensive and continuously updated databases of viral sequences, and to avoid biases towards detection of viral hits. The NCBI viral RefSeq database only contains reference genomes and is missing several types of human papillomaviruses (as well as more novel isolates for which official type numbers have not been assigned). After the finalization of the analysis, a reference viral database containing all (eukaryotic) viral sequences has been published [23], which unquestionably will facilitate future virome analysis. In virome studies viral findings are sometimes presented at genus or family level [24],[25],[26]. Assignment of contigs or reads to viral strains by BLAST or mapping is subject to some uncertainty, especially in cases where only shorter genome fragments are identified. While this approach prevents overestimation of the viral diversity identified, it also results in loss of information regarding the specific viral species or strains present, and impedes comparison of findings between studies. For these reasons, we have in the article presented the viruses identified in our study grouped at higher taxonomic levels as appropriate, while the details regarding specific strains identified by contig BLASTnx or read mapping is retained in the Supplementary Material.

RNA virus detection

Almost exclusively DNA viruses were detected in our study. Of RNA viruses, we detected human pegivirus A in five leukaemia samples (11%), a detection rate around 5 times higher than the prevalence of 2.2% in blood of healthy individuals in Denmark [27]. HIV-1 was found in an ascites sample from a colon cancer patient using retrovirus capture on a DNA library, implying that the virus exists as a provirus in this patient. Our virion enrichment protocol has proven successful in detecting RNA viruses in other studies [22],[28]. The low number of RNA viruses found here could

either reflect the absence of RNA viruses in the tissue types investigated, or limitations of the methods as outlined above. Mapping to RNA transcripts showed higher fractions of RNA-derived sequencing reads in total RNA sequencing compared to total DNA sequencing, though the increase was small for the POM121C transcript, confirming that the applied RNA sequencing methodology does target RNA sequences.

Large scale studies of viruses on human skin and other tissues of healthy individuals have mainly investigated DNA viruses [24],[25],[26],[29], whereas the RNA virome of healthy individuals at these tissue sites is not well characterised.

Enrichment methods characteristics

Among the applied enrichment methods vertebrate virus capture was the most sensitive, which complies with previous reports [30] and is expected, as it has the broadest specificity inherent to its design. Capture permits detection of divergent viral sequences with lower percent identity (based on the BLAST alignments) than what was identified here [2],[31]. Viral capture did not recover the novel HPV sequences detected by the other enrichment methods, which could be due to consistently low percent identity to the reference sequence throughout the genome, whereas more divergent anelloviruses detected by capture showed regionally higher (~90%) identity to the reference. Capturing divergent sequences can be achieved by lowering the stringency of the reaction [2],[32], which might have allowed detection of even more divergent viral sequences. For the other enrichment methods applied, the limitation for identification of low identity sequences lies in the BLAST algorithms, and we find these methods preferred for identification of more divergent viruses. Few additional viral contigs were identified by BLASTx, suggesting that the samples investigated mainly contain already known or more closely related viruses (and possibly very distantly related/unrelated novel viruses). High genome coverage was more often achieved for circular DNA enrichment compared to the other methods applied. This could indicate that phi29 polymerase-mediated amplification more frequently recovers larger, continuous genome fragments,

explaining why read mapping analysis only identified few additional virus-positive samples for this method.

Cross mapping of reads between closely related viral species

We found a few cases of samples showing detection of two closely related viral species, which we hypothesise to be artefacts arising due to sequence homology between the two species, rather than these samples truly being positive for both viruses.

The finding of a bladder cancer urine sample positive for both JCV (>10 million reads) and BKV (only 59 reads) led us to investigate the BKV reads further. Manual web-based BLAST (blastn algorithm) of the 59 BKV reads revealed that 56 of them had highest identity to JCV, while the remaining three showed highest identity to BKV. These results suggest that the majority of the BKV reads detected in this sample arise as a result of cross-mapping of reads originating from JCV but which map to BKV. The remaining three BKV reads could likely originate from bleedover of BKV reads from a BKV-positive sample sequenced on the same lane, showing higher BKV read count.

Likewise, samples positive for both HHV6A and 6B were investigated further. Manual BLAST of the reads mapping to HHV6B revealed highest identity to HHV6A for the two HHV6A and HHV6B-positive mycosis fungoides samples and a testicular cancer sample, while the opposite was the case for an oral cavity cancer sample. These findings suggest that reads mapping to one HHV6 species can occur as a result of cross mapping of reads originating from the other HHV6 species. The presence of contigs showing highest identity to HHV6A in the two high-coverage HHV6A samples supports the hypothesis of these samples containing HHV6A rather than HHV6B.

Such cross mapping of reads between BKV/JCV and HHV6A/6B can occur due to high local identity between the viral genomes. As is common practice, only one representative genome for each viral strain or species was included in the database used for mapping. Consequently, for closely related strains or species, reads originating from one virus might map to a different strain or

species, depending on their mutual sequence identity. Our assessment of such cross-mapping is based on re-BLAST of the reads in question, but further testing is necessary to confirm this hypothesis.

Merkel cell polyomavirus detection

MCPyV was only detected when applying virion enrichment DNA, not by circular DNA enrichment as could otherwise have been expected based on previous findings [24],[33]. This finding prompted us to analyse the MCPyV findings further. As the MCPyV genome was not included in the probe design of vertebrate virus capture, we were not able to confirm or disprove the results based on capture performed on the same samples. Non-enriched total DNA extracts from some of the MCPyV-positive samples were tested using real-time PCR with MCPyV-specific primers and were all negative (data not shown). Furthermore, the identified MCPyV sequences were remarkably similar between datasets, with practically all SNPs being shared across samples (data not shown). This finding is not expected if the sequences originate from true infections occurring in separate patients, and could thus suggest a possible contamination. Most of the MCPyV-positive samples were processed in sequential batches, which could indicate a commons source of the MCPyV sequences. It has been shown that MCPyV can be detected in swabs from environmental surfaces of both laboratories, private homes, and public spaces [34]. Based on these circumstances we suspect that MCPyV, in our study, is found as a result of contamination from e.g. laboratory surfaces, personnel, or reagents.

Bleedover between samples sequenced on the same lane

Bleedover of viral reads between datasets resulted in a number of false positives. Based on the applied threshold, 265 of a total of 1026 (26%) viral hits identified by mapping were considered bleedover. The viral read counts and coverage patterns from the mapping analysis proved important for evaluation of bleedover in the contig data. We observed cases of datasets showing high viral

read depth and full genome coverage by mapping, but for which *de novo* assembly had not succeeded in assembling the full viral genome. Consequently, short contigs in a dataset in the presence of longer contigs mapping to the same virus in other datasets from the same lane of the flow cell, were not necessarily indicative of bleedover. For example, for an HPV16 positive dataset having 15.9 million reads mapping to HPV16 we found 12 contigs with lengths between 203 and 851 nt, resulting in a total genome coverage of 37%. On the same lane, three other datasets, including a non-template control, all showed the full HPV16 genome assembled in 1 or 2 contigs, despite HPV16 read counts of only 8,450-13,817 reads, yielding a read count ratio of 0.05-0.08% relative to the high read count sample, and thus very likely represent bleedover and not true viral findings. Bleedover evaluation at contig level is manual and time consuming, however, failure to do so increases the risk of reporting false positives. Expectedly, the use of double indexing reduced the extent of bleedover, as previously shown [12].

Identification of viral sequences in non-template controls

In contrast to often practiced we included non-template controls with every round of sample processing, from extraction to library build and sequence analysis. Even though the non-template control libraries in most cases did not contain measurable DNA, they were sequenced and in all cases yielded sequencing reads (median 95,440; mean 6.9 millions). After investigation of the human viral hits, all viruses found at contig level could be explained by bleedover. However, read mapping detected viral reads, which were not removed by the applied bleedover threshold of 0.5%. These include reads mapping to MCPyV, HPVs, and anelloviruses. One possible explanation for the remaining reads is that the extent of bleedover often is higher in non-template control samples (likely applicable to other libraries with very low DNA content as well). An example of this is the finding of reads mapping to the novel HPV genome identified in a sample of mycosis fungoides in the non-template control sample sequenced on the same lane (sample NTC_s1383). The bleedover ratio for the HPV reads in this dataset was 6.9%, which is well above the otherwise observed level

in datasets from biological samples. The non-template controls also showed low level viral read counts, which could not readily be explained by a raised bleedover level, in some cases also reads mapping to viruses not occurring in other samples sequenced on the same lane. These findings are therefore not explained by bleedover, nor by sample cross-contamination. Other possible explanations could be contamination from previous sequencing runs or other sources, which are manifested to a higher extent in the non-template controls, due to low (or no) DNA actually being present in the generated libraries [35],[36].

References

1. Friis-Nielsen J, Kjartansdóttir KR, Mollerup S, Asplund M. Identification of Known and Novel Recurrent Viral Sequences in Data from Multiple Patients and Multiple Cancers. Viruses. **2016**; 8(2).
2. Vinner L, Mourier T, Friis-Nielsen J, et al. Investigation of Human Cancers for Retrovirus by Low-Stringency Target Enrichment and High-Throughput Sequencing. Scientific Reports. **2015**; 5:srep13201.
3. Mollerup S, Fridholm H, Vinner L, et al. Cutavirus in Cutaneous Malignant Melanoma. Emerg Infect Dis. **2017**; 23(2):363–365.
4. Lindgreen S. AdapterRemoval: easy cleaning of next-generation sequencing reads. BMC Research Notes. **2012**; 5:337.
5. Li H, Durbin R. Fast and accurate short read alignment with Burrows–Wheeler transform. Bioinformatics. **2009**; 25(14):1754–1760.
6. Morgulis A, Gertz EM, Schäffer AA, Agarwala R. A Fast and Symmetric DUST Implementation to Mask Low-Complexity DNA Sequences. Journal of Computational Biology. **2006**; 13(5):1028–1040.
7. Peng Y, Leung HCM, Yiu SM, Chin FYL. IDBA-UD: a de novo assembler for single-cell and metagenomic sequencing data with highly uneven depth. Bioinformatics. **2012**; 28(11):1420–1428.
8. Altschul SF, Gish W, Miller W, Myers EW, Lipman DJ. Basic local alignment search tool. Journal of Molecular Biology. **1990**; 215(3):403–410.
9. Langmead B, Salzberg SL. Fast gapped-read alignment with Bowtie 2. Nat Meth. **2012**; 9(4):357–359.

10. Buchfink B, Xie C, Huson DH. Fast and sensitive protein alignment using DIAMOND. Nat Meth. **2015**; 12(1):59–60.

11. Krzywinski M, Schein J, Birol I, et al. Circos: an information aesthetic for comparative genomics. Genome Res. **2009**; 19(9):1639–1645.

12. Kircher M, Sawyer S, Meyer M. Double indexing overcomes inaccuracies in multiplex sequencing on the Illumina platform. Nucleic Acids Res. **2012**; 40(1):e3.

13. Nistelberger HM, Smith O, Wales N, Star B, Boessenkool S. The efficacy of high-throughput sequencing and target enrichment on charred archaeobotanical remains. Scientific Reports. **2016**; 6:srep37347.

14. R Core Team. R: A language and environment for statistical computing. Vienna, Austria: R Foundation for Statistical Computing; 2015. Available from: http://www.R-project.org/

15. Sarkar D. Lattice: Multivariate Data Visualization with R. New York: Springer; 2008. Available from: http://lmdvr.r-forge.r-project.org

16. Kumar S, Stecher G, Tamura K. MEGA7: Molecular Evolutionary Genetics Analysis Version 7.0 for Bigger Datasets. Mol Biol Evol. **2016**; 33(7):1870–1874.

17. Caracausi M, Piovesan A, Antonaros F, Strippoli P, Vitale L, Pelleri MC. Systematic identification of human housekeeping genes possibly useful as references in gene expression studies. Mol Med Rep. **2017**; 16(3):2397–2410.

18. Tang K-W, Alaei-Mahabadi B, Samuelsson T, Lindh M, Larsson E. The landscape of viral expression and host gene fusion and adaptation in human cancer. Nature Communications. **2013**; 4:ncomms3513.

19. Strong MJ, Blanchard E, Lin Z, et al. A comprehensive next generation sequencing-based virome assessment in brain tissue suggests no major virus - tumor association. Acta Neuropathol Commun. **2016**; 4(1):71.

20. Cao S, Strong MJ, Wang X, et al. High-Throughput RNA Sequencing-Based Virome Analysis of 50 Lymphoma Cell Lines from the Cancer Cell Line Encyclopedia Project. J Virol. **2014**; 89(1):713–729.

21. Asplund M, Kjartansdóttir KR, Mollerup S, et al. Contaminating viral sequences in high-throughput sequencing viromics: a linkage study of 700 sequencing libraries. Clin Microbiol Infect. **2019**.

22. Jensen RH, Mollerup S, Mourier T, et al. Target-Dependent Enrichment of Virions Determines the Reduction of High-Throughput Sequencing in Virus Discovery. PLOS ONE. **2015**; 10(4):e0122636.

23. Goodacre N, Aljanahi A, Nandakumar S, Mikailov M, Khan AS. A Reference Viral Database (RVDB) To Enhance Bioinformatics Analysis of High-Throughput Sequencing for Novel Virus Detection. mSphere. **2018**; 3(2).

24. Foulongne V, Sauvage V, Hebert C, et al. Human skin microbiota: high diversity of DNA viruses identified on the human skin by high throughput sequencing. PLoS ONE. **2012**; 7(6):e38499.

25. Wylie KM, Mihindukulasuriya KA, Zhou Y, Sodergren E, Storch GA, Weinstock GM. Metagenomic analysis of double-stranded DNA viruses in healthy adults. BMC Biology. **2014**; 12:71.

26. Hannigan GD, Meisel JS, Tyldsley AS, et al. The Human Skin Double-Stranded DNA Virome: Topographical and Temporal Diversity, Genetic Enrichment, and Dynamic Associations with the Host Microbiome. mBio. **2015**; 6(5):e01578-15.

27. Christensen PB, Fisker N, Mygind LH, et al. GB Virus C epidemiology in denmark: Different routes of transmission in children and low- and high-risk adults. J Med Virol. **2003**; 70(1):156–162.

28. Hansen TA, Mollerup S, Nguyen N, et al. High diversity of picornaviruses in rats from different continents revealed by deep sequencing. Emerg Microbes Infect. **2016**; 5(8):e90.

29. Oh J, Byrd AL, Deming C, Conlan S, Kong HH, Segre JA. Biogeography and individuality shape function in the human skin metagenome. Nature. **2014**; 514(7520):59–64.

30. Briese T, Kapoor A, Mishra N, et al. Virome Capture Sequencing Enables Sensitive Viral Diagnosis and Comprehensive Virome Analysis. mBio. **2015**; 6(5):e01491-15.

31. Wylie TN, Wylie KM, Herter BN, Storch GA. Enhanced virome sequencing using targeted sequence capture. Genome Res. **2015**; 25(12):1910–1920.

32. Mason VC, Li G, Helgen KM, Murphy WJ. Efficient cross-species capture hybridization and next-generation sequencing of mitochondrial genomes from noninvasively sampled museum specimens. Genome Res. **2011**; 21(10):1695–1704.

33. Schowalter RM, Pastrana DV, Pumphrey KA, Moyer AL, Buck CB. Merkel Cell Polyomavirus and Two Novel Polyomaviruses Are Chronically Shed from Human Skin. Cell Host Microbe. **2010**; 7(6):509–515.

34. Foulongne V, Courgnaud V, Champeau W, Segondy M. Detection of Merkel cell polyomavirus on environmental surfaces. J Med Virol. **2011**; 83(8):1435–1439.

35. Lusk RW. Diverse and widespread contamination evident in the unmapped depths of high throughput sequencing data. PLoS ONE. **2014**; 9(10):e110808.

36. Salter SJ, Cox MJ, Turek EM, et al. Reagent and laboratory contamination can critically impact sequence-based microbiome analyses. BMC Biology. **2014**; 12:87.
