# Supplementary figures and images for: High-Throughput Sequencing-Based Investigation of Viruses in Human Cancers by Multienrichment Approach
Source: J Infect Dis. 2019 Jun 28;220(8):1312–24. doi: 10.1093/infdis/jiz318 (PMC6743825; doi:10.1093/infdis/jiz318)

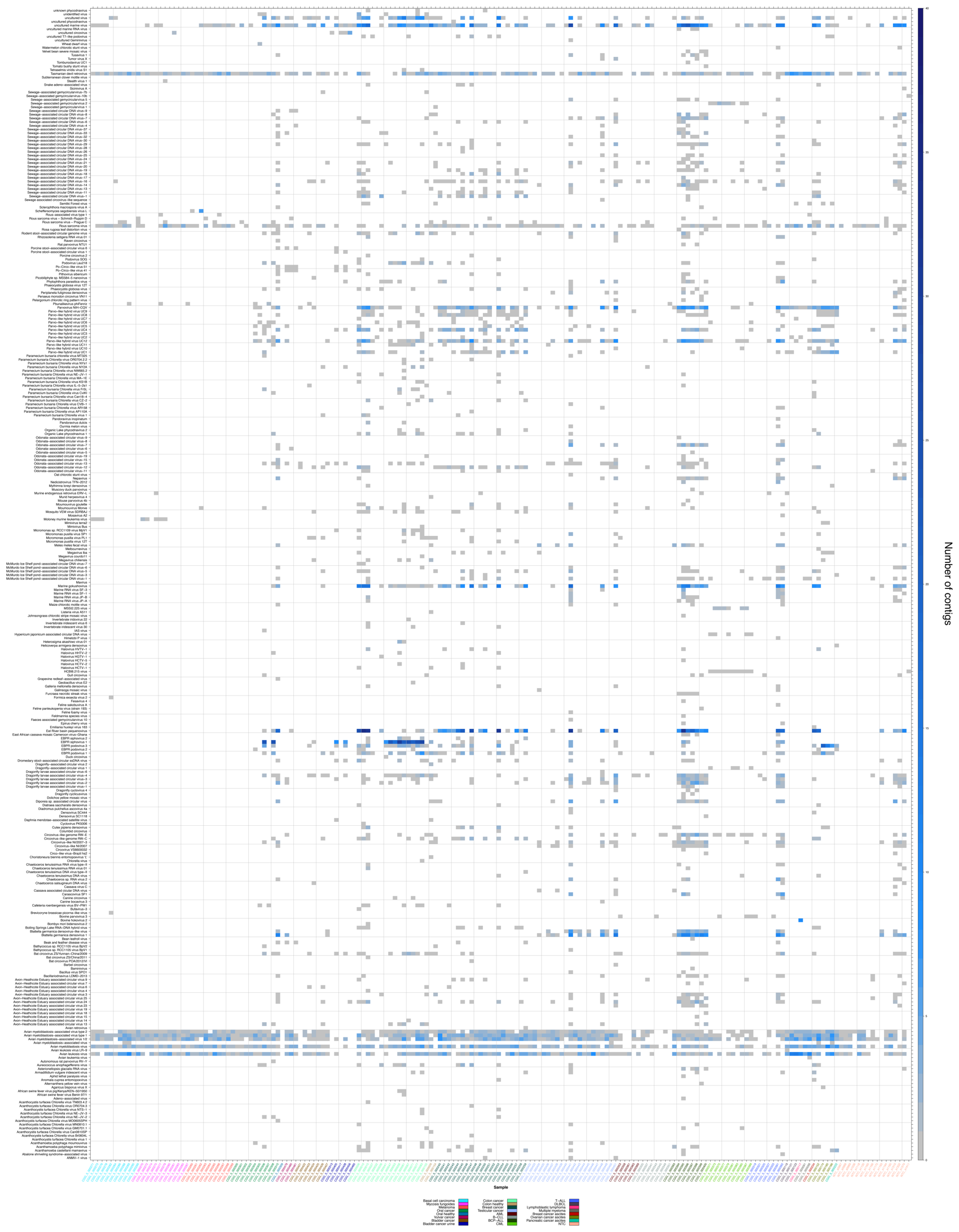

Supplement: jiz318_Suppl_Supplementary_Figure_S6 [file jiz318_suppl_supplementary_figure_s6.pdf]

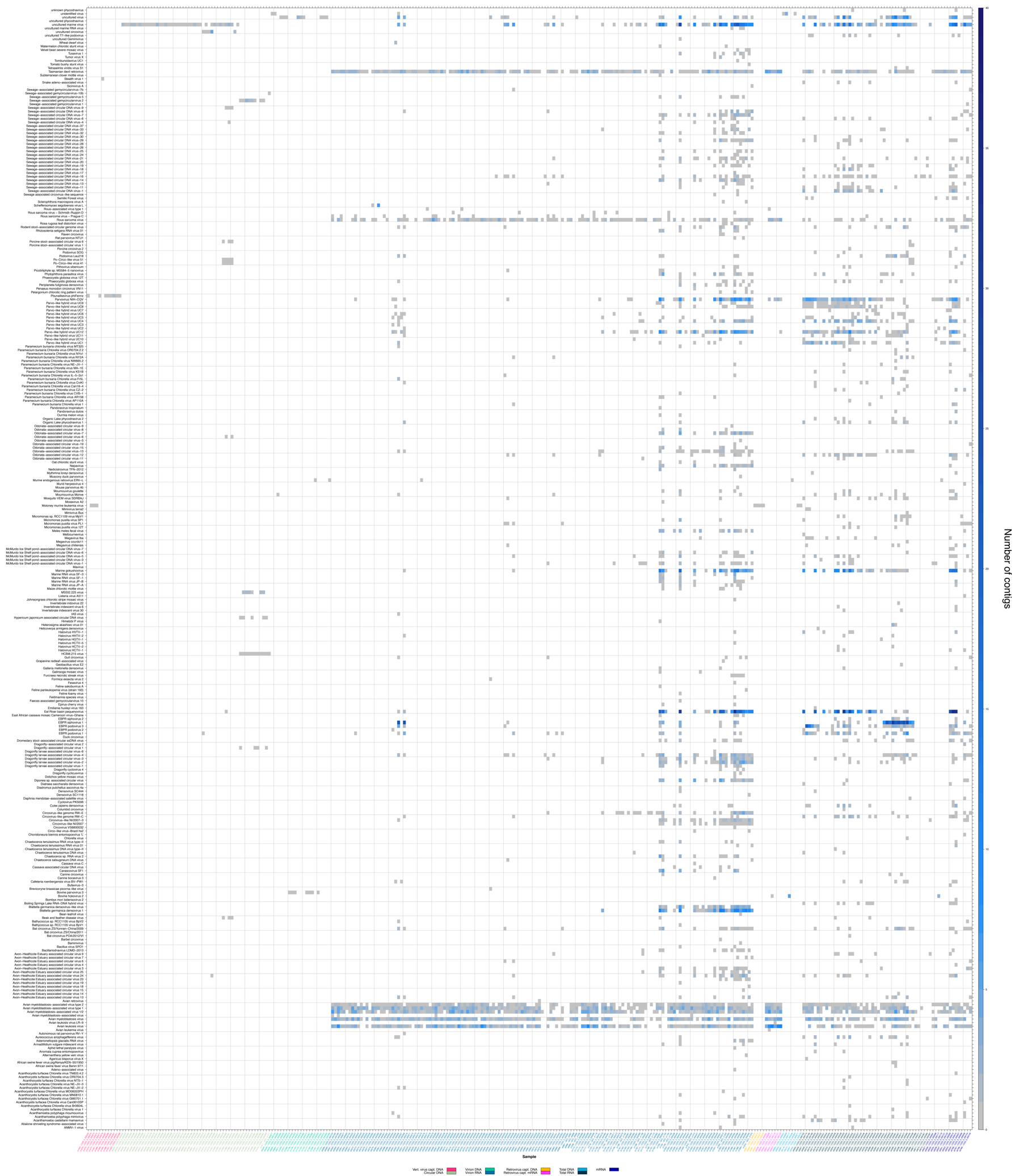

Supplement: jiz318_Suppl_Supplementary_Figure_S7 [file jiz318_suppl_supplementary_figure_s7.pdf]
